# Calpain-1: a Novel Antiviral Host Factor Identified in Porcine Small Intestinal Mucus

**DOI:** 10.1128/mbio.00358-22

**Published:** 2022-09-14

**Authors:** Yuchen Li, Xiuyu Wang, En Zhang, Ruiling Liu, Chengjie Yang, Ying Duan, Yuqi Jiang, Qian Yang

**Affiliations:** a MOE Joint International Research Laboratory of Animal Health and Food Safety, College of Veterinary Medicine, Nanjing Agricultural University, Nanjing, Jiangsu, China; University of Maryland; Washington University School of Medicine

**Keywords:** small intestinal mucus, Muc2, calpain-1, antiviral activity, goblet cells

## Abstract

The thick mucus layer covering of the intestinal epithelium has received increasing attention, owing to its protective role in intestinal infection. However, the exact mechanisms by which the mucus increases intestinal resistance against viral infection remain largely unclear. Here, we identify prominent antiviral activity of the small intestinal mucus and extracted total mucus proteins, as evidenced by their inhibitory effects against porcine epidemic diarrhea virus (PEDV) infection. Of all the extracted mucus proteins, mucin 2 and fraction III (~70 kDa) exhibited potent antiviral activity. We further evaluated the antiviral effects of three candidate factors in fraction III and found that calpain-1 contributed substantially to its antiviral activity. *In vivo* studies demonstrated that oral administration of calpain-1 provided effective protection against intestinal PEDV infection. As a calcium-activated cysteine protease, calpain-1 inhibited viral invasion by binding to and hydrolyzing the S1 domain of the viral spike protein. The region between amino acids 297 and 337 in the b domain of PEDV S1 protein was critical for calpain-1-mediated hydrolysis. Further investigation indicated that calpain-1 could be produced by goblet cells between intestinal epithelia. Taken together, the results of our study revealed calpain-1 to be a novel antiviral protein in porcine small intestinal mucus, suggesting that calpain-1 has potential for defending against intestinal infections.

## INTRODUCTION

The epithelial mucus layer of the intestine constitutes the first line of defense against intestinal infection (1, 2). The broad range of its protective functions, especially its antibacterial and antiviral activities, has aroused widespread interest in the intestinal mucus layer (3–5). In addition to functioning as a physical barrier, the intestinal mucus layer can attenuate or inactivate pathogenic microorganisms via defense molecules (6, 7). Although some progress has been made in elucidating the protective role of the intestinal mucus layer, the functions and underlying mechanisms of its protective molecules remain mostly unknown.

The well known dense and reticular features of mucus are highly dependent on a variety of mucin molecules, which contribute to the physical protection conferred by the mucus barrier (5). In the small intestine, the main secreted mucus protein is mucin 2 (Muc2), which comprises more than 5,100 amino acids and has a molecular weight of approximately 2.7 MDa. This gel-forming glycoprotein forms the organizational structure of mucus and provides protection against intestinal infection by pathogenic bacteria (8). For example, Muc2-knockout mice exhibit a collapsed mucus layer and increased susceptibility to Salmonella enterica and Citrobacter rodentium infection (9, 10). Muc2 can exert antibacterial effects via multiple mechanisms of action, including binding, blocking, or attenuating pathogenic bacteria, as well as activating a robust innate immune response (11, 12). Mucins limit pathogen binding to epithelial cells not only via steric hindrance or by acting as releasable decoys for microbial adhesions (13) but also by inhibiting the expression of the bacterial virulence genes of Pseudomonas aeruginosa via their glycan complexity (14). Altered mucin structure and increased Muc2 production have been observed in intestinal viral infection, such as astrovirus (15), rotavirus (16), and transmissible gastroenteritis virus (TGEV) infections (17), generating widespread interest in its potential role in viral infection resistance. Currently, little is known about the protective effects of other active molecules in the intestinal mucus compared to those of the mucin family. Although some active molecules, such as TFF1, CLCA1, FCGBP, and ZG16, have been identified in the intestinal mucus, their functions are mainly related to regulation of mucus secretion, enterocyte renewal, and interactions with commensal bacteria (18). Therefore, the identification of novel protective components in the intestinal mucus might aid in the development of novel antiinfective substances.

Accumulating evidence has demonstrated that the small intestinal mucosa is a major invasion and infection site for the vast majority of intestinal pathogens, including pathogenic enterobacteria (such as Salmonella, Campylobacter jejuni, Yersinia enterocolitica, and enterotoxigenic Escherichia coli) (19, 20) and enteric viruses (such as enteroviruses, noroviruses, rotaviruses, astroviruses, and various swine enteric coronaviruses) (21, 22). The potential protective role of the small intestinal mucus deserves particular attention. Many viruses with enteric tropism can target the small intestinal mucosa (23–26), including the notorious enteric coronaviruses that cause porcine diarrhea. Among them, the porcine epidemic diarrhea virus (PEDV) has caused substantial economic loss to the global pig industry (21, 27, 28). PEDV primarily targets small intestinal epithelium and causes superficial villous enterocyte necrosis, leading to mortality rates as high as 100% in neonatal piglets. To date, no vaccine has provided complete mucosal immune protection against PEDV-induced intestinal infection (28). Accordingly, enhancing the intestinal mucus barrier to limit viral contact with intestinal epithelial cells is an important strategy to control PEDV infection. Although porcine gastric mucin exert broad-spectrum antiviral effects by restricting viral diffusion (29), the precise protective role and underlying mechanisms of the porcine small intestinal mucus are not well understood. Therefore, this study aimed to explore the effects of porcine small intestinal mucus against PEDV infection and identify the potential protective molecules and their underlying mechanisms.

## RESULTS

### Mucus secretion exhibits age-dependent changes in porcine small intestinal mucosa.

To compare the mucus secretion characteristics of the small intestine in newborn and weaned piglets, we first investigated the thickness of the porcine small intestinal mucus. The jejunal ligated loops of newborn and weaned piglets were injected with fluorescent latex beads, which were expelled by the mucus layer and converged into a line. Using confocal laser scanning microscopy (CLSM), the thickness of the mucus layer was defined as the distance between the observed line and the intestinal epithelium. The small intestinal mucus was significantly thicker in weaned piglets than in newborn piglets (Fig. S1A). The markedly upregulated mRNA expression of goblet cell markers *Retnlb* and *Tff1*, as well as mucins *Muc1*, *Muc2*, and *Muc4* (30), further illustrated the distinct mucus secretory function in the small intestine of weaned piglets (Fig. S1B to F). Furthermore, Periodic acid-Schiff (PAS) staining showed that the number of mucus-secreting cells increased with the age of the pig, which demonstrated a characteristic age-dependent distribution in the small intestinal mucosal (Fig. S2A and B). The crude mucus of the small intestine was isolated from the jejunum of finisher pigs (260 days old) and used to detect antiviral activity (Fig. S2C).

10.1128/mbio.00358-22.1 FIG S1 Comparison of mucus layer thickness and mRNA expression levels of goblet cell markers in small intestine of newborn and weaning piglets. (A) Mucus thickness measured in five small intestinal tissues of newborn and weaned piglets. Representative images of DyLight 594-labeled latex bead distribution in the small intestine of newborn and weaned piglets are presented. The gap between fluorescently labeled beads and the epithelium was considered the mucus thickness. Bar = 25 μm. (B to F) The relative mRNA expression levels of goblet cell markers, including Muc1 (B), Muc2 (C), Muc4 (D), *Retnlb* (E), and TFF1 (F), were quantified via RT-qPCR. All data are presented as means ± standard deviation (SD). Analysis of variance (ANOVA) was used to analyze differences between three or more groups, and *t* tests were used to analyze differences between two groups. The data presented are from at least three independent experiments. *, *P < *0.05; **, *P < *0.01; ***, *P < *0.001. Download FIG S1, PDF file, 0.3 MB. Copyright © 2022 Li et al. 2022 Li et al. https://creativecommons.org/licenses/by/4.0/ This content is distributed under the terms of the Creative Commons Attribution 4.0 International license.

10.1128/mbio.00358-22.2 FIG S2 The distribution of goblet cells in small intestine of pig at different age. (A) Goblet cells in the jejunal and ileal mucosa are shown by AB/PAS staining. (B) Quantification of goblet cells randomly evaluated in 10 villus-crypt units in each of the three individual sections, summarized in a column plot. (C) Schematic of porcine small intestinal mucus collection. All data are presented as means ± SD. ANOVA was used to analyze differences between three or more groups, and *t* tests were used to analyze the differences between two groups. The results are from at least three independent experiments. *, *P < *0.05; **, *P < *0.01; and ***, *P < *0.001. Download FIG S2, PDF file, 0.3 MB. Copyright © 2022 Li et al. 2022 Li et al. https://creativecommons.org/licenses/by/4.0/ This content is distributed under the terms of the Creative Commons Attribution 4.0 International license.

### Protective role of intestinal mucus proteins against PEDV infection.

**FIG 1:**
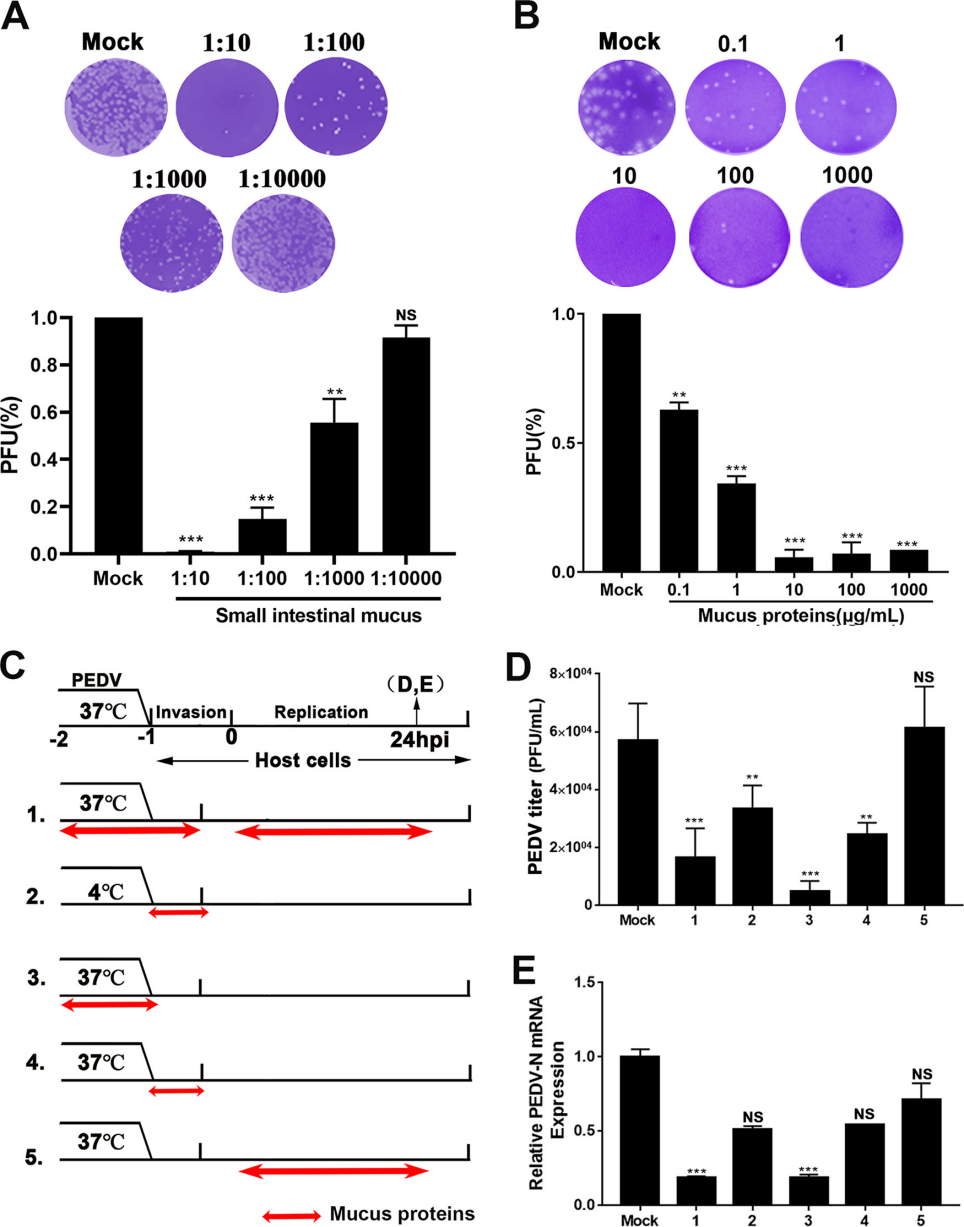
Influence of small intestinal mucus on porcine epidemic diarrhea virus (PEDV) infection. (A, B) Antiviral effect of the small intestinal mucus (A) and total mucus proteins (B) against PEDV determined using a plaque reduction assay. The isolated intestinal mucus and extracted mucus proteins were diluted and tested against live PEDV. Neutralization activity was evaluated. The results are representative of at least three independent experiments, and each time, the mucus was isolated from three different pigs (healthy littermates), with three technical replicates of mucus samples per pig. (C) Analysis of the viral stage at which the total mucus proteins exert anti-PEDV effects. Before inoculation on Vero E6 cells, PEDV was treated with mucus proteins at 37 or 4°C for 1 h. Mucus was added at the replication phase of PEDV (group 5). Double-headed red arrows indicate the presence of reagent. Experimental groups are labeled using numbers on the left. (D) Infectious particles in the culture medium evaluated through plaque formation and quantified in the histogram. The numbers below the graph correspond to the group numbers in panel B. (E) The number of RNA copies of PEDV was quantified using reverse transcription-quantitative PCR (RT-qPCR). All data are shown as means ± standard deviation (SD), and the comparisons were performed by one-way analysis of variance (ANOVA). Results are from at least three independent experiments. *, *P < *0.05; **, *P < *0.01; ***, *P < *0.001; NS, not significant; PEDV-N, PEDV nucleocapsid; PFU, plaque forming unit.

The plaque reduction assay was used to preliminarily evaluate the antiviral function of porcine small intestinal mucus. Pretreatment with mucus resulted in a significant reduction in viral plaque formation, even at a 1,000-fold dilution (Fig. 1A). To determine the viral stage of infection at which mucus exerted an anti-PEDV effect, as well as its possible mode of action, bovine serum albumin (BSA, negative control), mucus, or mucus treated by enzymolysis or thermal denaturation was added to the virus or host cells before or during infection (Fig. S3A to D). Upon incubation with mucus for 1 h at 37°C or 4°C, the progeny virion yield and viral replication were substantially reduced. The prominent difference between group 1 (virus incubated with mucus at 37°C) and group 2 (mucus applied during viral replication) indicated that the inhibitory effect declined when mucus was added during the viral replication phase. However, heat-denatured mucus retained partial antiviral activity, suggesting that the polymer structure blocked viral diffusion. A reduced inhibitory effect was also detected when protease-treated mucus was added before PEDV inoculation compared to after inoculation. These results indicated that the inhibitory effect of porcine small intestinal mucus against PEDV was related to its physical barrier function and relied on protein components with antiviral activity. The result of the Cell Counting kit 8 (CCK-8) assay showed that the crude mucus concentration did not affect cell viability (Fig. S3E).

10.1128/mbio.00358-22.3 FIG S3 Influence of small intestinal mucus on porcine epidemic diarrhea virus (PEDV) infection. (A) Analysis of the stage at which mucus exerts its anti-PEDV effect. Before inoculation on Vero E6 cells, PEDV was treated with mucus proteins at 37 or 4°C for 1 h, or it was incubated with heat-denatured and hydrolyzed mucus at 37°C for 1 h. The mucus was added at the replication phase of PEDV (group 2). Double-headed red arrows indicate the presence of the reagent. The experimental groups are labeled with numbers on the left. The culture medium and cell samples were harvested at 24 hours postinfection (hpi). (B) Infectious particles in the culture medium were evaluated through plaque formation assay. The histogram summarizes the plaque assay results. (C) Nucleoprotein of PEDV was detected through Western blot analysis. A histogram of band intensities is shown. (D) Number of copies of the RNA genome of PEDV was quantitated via RT-qPCR. (E) Vero E6 cells were incubated with different concentrations of small intestinal mucus for 24 h and then evaluated using a CCK8 assay. (F) Adding different concentrations of the total intestinal mucus proteins on Vero E6 cells for 24 h to determine cell viability by a CCK8 proliferation assay. All data are presented as means ± SD from at least three independent experiments, and comparisons were performed using one-way ANOVA. *, *P < *0.05; **, *P < *0.01; ***, *P < *0.001. Download FIG S3, PDF file, 0.2 MB. Copyright © 2022 Li et al. 2022 Li et al. https://creativecommons.org/licenses/by/4.0/ This content is distributed under the terms of the Creative Commons Attribution 4.0 International license.

Based on the above results, the total proteins were extracted and evaluated for their antiviral activity. As determined by the CCK-8 proliferation assay, the total mucus proteins also had no effect on cell viability, even at a concentration of 1 mg/mL (Fig. S3F). However, the total mucus proteins suppressed viral plaque formation in a dose-dependent manner (Fig. 1B). To determine the viral stage of infection at which the total mucus proteins exerted an anti-PEDV effect, BSA (negative control) or the total mucus proteins were added to the virus or cells at different times before or during infection. The infection process was divided into virus adsorption (4°C), preinfection (37°C), viral invasion (1 h postinfection [hpi] at 37°C), and viral replication (1 to 24 hpi) phases (Fig. 1C). Viral titers were determined in the culture medium, and viral RNA levels were evaluated in Vero E6 cells at 24 hpi (Fig. 1D and E). BSA had no significant effect at any viral stage, and the results for BSA supplementation at the different phases were averaged and used as control values. Similar to the antiviral action of intestinal mucus, the total mucus proteins exerted antiviral effects during the viral invasion stage, especially when directly incubated with the virus. No antiviral effects were observed when the total mucus proteins were added at the viral replication stage. These results suggested that the mucus proteins may act directly on PEDV particles, causing a loss of infectivity.

### Antiviral activity of mucus proteins with different molecular weights.

**FIG 2:**
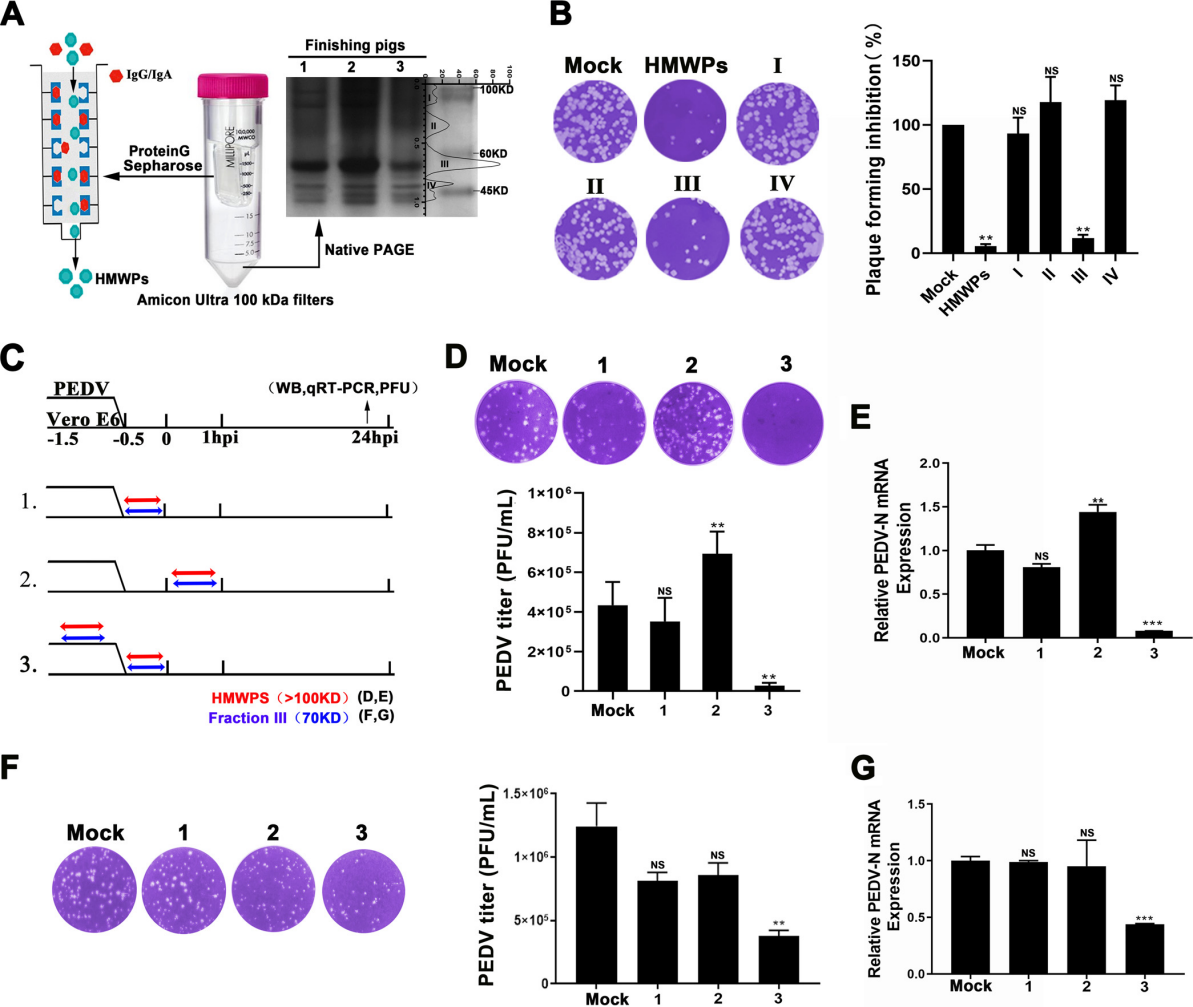
Characterization of proteins with antiviral activity in the small intestinal mucus. (A) Proteins in porcine small intestinal mucus were divided into proteins with molecular weights greater than (HMWPs) and less than (LMWPs) 100 kDa. LMWPs were further divided into four fractions (I to IV). (B) Antiviral activity of purified HMWPs and fractions I to IV determined using plaque reduction assay. The protein concentration used was 10 μg/mL. (C) Schematic diagram of identification of viral stage-dependent effects of purified HMWPs and fraction III against PEDV infection. Double-headed red arrows indicate the presence of reagent; blue indicates purified HMWPs; and red indicates mucus fraction III. (D to G) Infectious particles in the culture medium treated with HMWPs (D) or mucus fraction III (F) determined using plaque formation assay. The histogram summarizes the plaque assay results; numbers below the graph correspond to the numbers in panel C. PEDV mRNA levels in Vero E6 cells from groups treated with purified HMWPs (E) or mucus fraction III (G). The Western blotting results were evaluated based on band intensity, and the RNA levels were quantified via RT-qPCR. All data are presented as means ± SD, and comparisons were performed by one-way ANOVA. The results are from at least three independent experiments. *, *P < *0.05; **, *P < *0.01; ***, *P < *0.001; HMWP, high-molecular-weight protein; LMWP, low-molecular-weight protein; hpi, hours postinfection; WB, Western blotting; PEDV-N, PEDV Nucleocapsid; PFU, plaque forming unit.

Mucus proteins were separated into high-molecular-weight proteins (HMWPs) (>100 kDa), which were collected and purified through protein G affinity chromatography, and low-molecular-weight proteins (LMWPs) (<100 kDa), which were divided into four protein fractions (I to IV) that were purified and collected through electroelution (Fig. 2A). The antiviral activity of the purified HMWPs and the four LMWP fractions were detected using the plaque reduction assay. Both the HMWPs and proteins in fraction III exhibited significant antiviral activity (Fig. 2B). Furthermore, we evaluated the addition of HMWPs and proteins in fraction III at different stages of viral infection (Fig. 2C). Pretreatment with HMWPs reduced viral protein and mRNA expression levels, as well as the progeny virion yield, in PEDV-infected Vero E6 cells (Fig. 2D and E; Fig. S4A). However, HMWPs supplementation in the viral replication phase increased infectious particle release and viral mRNA levels (Fig. 2D and E).

10.1128/mbio.00358-22.4 FIG S4 Antiviral activity of Muc2 in the gastrointestinal tract of pigs. (A) Identification of viral stage-dependent effects of purified HMWPs against PEDV infection. A similar experimental design is described in legend to Fig. 2C. PEDV protein levels in Vero E6 cells from groups treated with purified HMWPs were determined. (B) Protein profile analysis of the high-molecular-weight proteins (HMWPs) in mucus proteins. (C) Muc2 (1 mg/mL) was added during invasion (group 1) or replication phase (group 2) of PEDV infection. Before inoculation on Vero E6 cells, PEDV was treated with Muc2 at 37°C for 1 h (group 3). Double-headed red arrows indicate the presence of the reagent. At 24 hpi, cell samples were harvested to determine the protein expression of PEDV in Vero E6 cells. (D) Infectious particles in the culture medium were detected using a plaque formation assay. The histogram summarizes the plaque assay results. The numbers below the graph correspond to the numbering of experiments in panel C. (E) The number of copies of the RNA genome of PEDV was quantitated via RT-qPCR. (F, G) Prior to the assays determining the antiviral activity of purified HMWPs, the protein mixture was pretreated with blocking antibodies against Muc2 with a certain concentration gradient. The viral titers in the supernatant (F) and the production of viral RNA (G) in epithelial cells from different groups were detected. All data are presented as means ± SD from at least three independent experiments; comparisons were performed using one-way ANOVA. *, *P < *0.05; **, *P < *0.01; ***, *P < *0.001. Download FIG S4, PDF file, 0.2 MB. Copyright © 2022 Li et al. 2022 Li et al. https://creativecommons.org/licenses/by/4.0/ This content is distributed under the terms of the Creative Commons Attribution 4.0 International license.

As Muc2 is the main component of the nonstructural extracellular proteins of HMWPs, its protective role against PEDV infection was further explored (Fig. S4B). Similar to the direct antiviral effect of the HMWPs, pretreatment with Muc2 significantly suppressed viral replication and infectious particle release. However, PEDV replication and release were not affected by the addition of Muc2 at the viral replication phase (Fig. S4C to E). In addition, pretreatment with anti-Muc2 antibody abolished the antiviral activity of HMWPs in a dose-dependent manner, indicating that its antiviral activity was largely dependent on Muc2 (Fig. S4F and G). The results of the CCK-8 assay showed that the concentration of Muc2 (1 mg/mL) used did not noticeably affect the viability of epithelial cells (Fig. S5A). Moreover, fraction III exhibited prominent antiviral activity without cytotoxicity (Fig. S5B), which could efficiently inhibit PEDV infection by pretreating with virions (Fig. 2F and G; Fig. S5C). Therefore, the specific functional molecules in fraction III warranted further investigation.

10.1128/mbio.00358-22.5 FIG S5 Assessment of the cytotoxicity of Muc2 and mucus protein fraction III against Vero E6 cells. (A) Cell viability was determined by CCK-8 assay after treatment of the Vero E6 cells with different concentrations of Muc2 for 24 h. (B) After treatment with different concentration of protein fraction III for 24 h, the cell viability of Vero E6 cells were detected by CCK-8 assay. (C) Identification of viral stage-dependent effects of fraction III against PEDV infection. Similar experimental designs were described in Fig. 2C. PEDV protein in Vero E6 cells from groups treated with mucus fraction III were determined. (D) Following incubation with the purified protein in fraction III, Vero E6 cells were inoculated with PEDV. Anti-PEDV effects were determined by Western blotting. “Mock” represents the group treated with BSA (100 μg/mL). All data are the means ± SD, comparisons performed with one-way ANOVA. *, *P < *0.05; **, *P < *0.01; ***, *P < *0.001. The results are from at least three different experiments. CCK-8, Cell Counting kit 8. Download FIG S5, PDF file, 0.2 MB. Copyright © 2022 Li et al. 2022 Li et al. https://creativecommons.org/licenses/by/4.0/ This content is distributed under the terms of the Creative Commons Attribution 4.0 International license.

### Calpain-1 plays a crucial role in the antiviral activity of proteins in fraction III.

**FIG 3:**
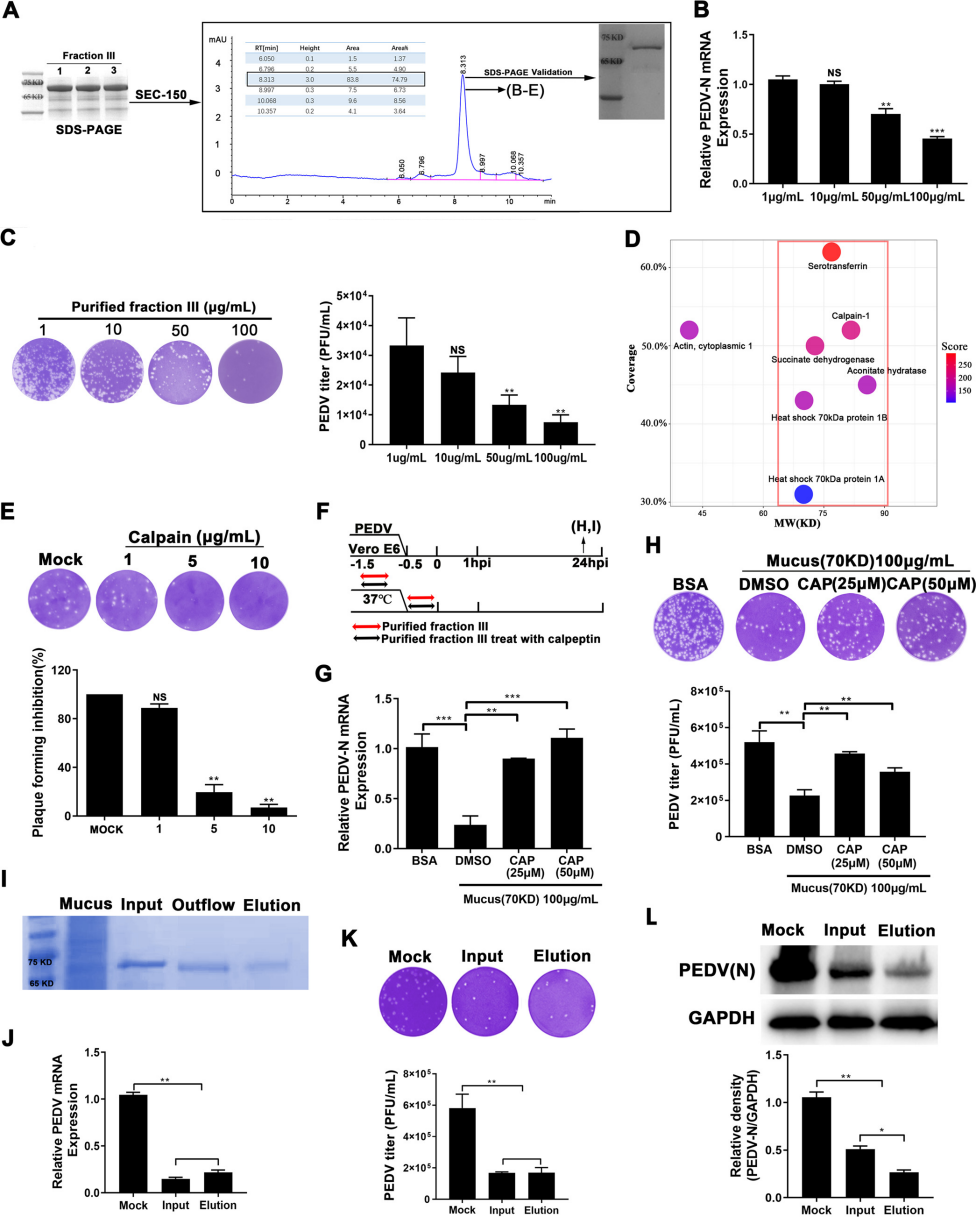
Calpain-1 mediates antiviral effects of protein fraction III. (A) Mucus protein fraction III was prepared using nondenaturing PAGE, collected through electroelution and purified using size-exclusion chromatography. Lanes 1 to 3 represent three replicates of fraction III. Shown is a chromatographic profile of fraction III in mucus obtained using an AKTA Purifier system with a SEC-150 gel-filtration column. The main peaks accounting for 74.79% of the total area were collected and verified with respect to molecular weight to obtain the purified protein fraction III. (B, C) Following incubation with the purified protein in fraction III, Vero E6 cells were inoculated with PEDV. Anti-PEDV effects were evaluated via Western blotting (B) and plaque reduction assay (C). (D) After verifying its antiviral activity, liquid chromatography-tandem mass spectrometry (LC-MS/MS) was used to determine the amino acid sequence of the purified protein in fraction III. The matched proteins, based on peptide fragment information, are shown in a bubble diagram. The protein molecular weight is shown on the *x* axis, the percentage of protein coverage is shown on the *y* axis, and the color of the dot represents the score. (E) Plaque formation ability following pretreatment with calpain-1 (0 to 10 μg) for 1 h. (F) Antiviral effect of the purified protein in fraction III treated with calpain-1 inhibitor for 1 h. (G, H) Cellular viral RNA levels for different groups (G) and viral titers (H) in medium quantified using RT-qPCR and plaque assay, respectively. (I) Purification of calpain-1 from the purified protein in fraction III using affinity chromatography. SDS-PAGE analysis of purified calpain-1. “Input” indicates the purified fraction III, “Outflow” indicates the flowthrough, “Elution” indicates the purified calpain-1. (J to L) mRNA expression (J), virus titer (K), and cellular protein (L) of PEDV in the culture supernatant of Vero E6 cells inoculated with PEDV pretreated with the input proteins (fraction III, 10 μg/mL) and the elution proteins (purified calpain-1, 10 μg/mL). The data are presented as means ± SD of three independent experiments. *, *P < *0.05; **, *P < *0.01; and ***, *P < *0.001. BSA, bovine serum albumin; CAP, calpain-1; PEDV-N, PEDV nucleocapsid.

Fraction III of the LMWPs was further purified through size-exclusion chromatography (Zenix SEC-150). The absorption peak of proteins that were approximately 70 kDa in size was detected at a retention time of 8.313 s and accounted for nearly 74.79% of proteins in fraction III. Sodium dodecyl sulfate-polyacrylamide gel electrophoresis (SDS-PAGE) was used to verify the molecular weight of the protein purified in fraction III, which migrated with an apparent molecular mass of 70 kDa (Fig. 3A). The plaque formation assay demonstrated that pretreatment with this purified protein significantly decreased PEDV titers in a dose-dependent manner; 100 μg/mL purified protein in fraction III reduced PEDV infection by 70% and induced consistent changes in viral protein and RNA levels (Fig. 3B and C; Fig. S5D).

The purified protein in fraction III was further analyzed through liquid chromatography-tandem mass spectrometry (LC-MS/MS), revealing 873 peptide fragments from 142 proteins identified in the UniProt-Sus database. High-scoring proteins with a molecular weight of approximately 70 kDa were identified according to the peptide coverage (Fig. 3D, red frame). Among the candidate proteins, three secretory proteins, serotransferrin, calpain-1, and heat shock 70 kDa (HSP70), were chosen for further analysis based on their potential antiviral function. Preincubation with transferrin and HSP70 did not exert direct anti-PEDV effects (Fig. S6A to F); however, calpain-1 reduced viral plaque formation by 80% (Fig. 3E), which was similar to the anti-PEDV effect of fraction III, even at a 10-fold lower dosage. The concentration of transferrin and HSP70 used did not induce cytotoxicity (Fig. S6G and H). Additionally, treatment with calpeptin, an inhibitor of calpain-1, abrogated the antiviral effect of the purified protein in fraction III (Fig. 3F) and attenuated the effect on viral mRNA levels (Fig. 3G) and infectious virion release (Fig. 3H). No changes in cell viability were observed following treatment with 10 μg/mL calpain-1 (Fig. S6I) and 500 mM calpeptin (Fig. S6J), indicating that cytotoxicity was not responsible for the observed effects.

10.1128/mbio.00358-22.6 FIG S6 Effect of transferrin or HSP70 treatment on PEDV infection. PEDV was incubated with various concentrations of transferrin or HSP70 at 37°C for 1 h, and the mixture was inoculated on Vero E6 cells at 37°C for 1 h. Then the inoculum and unattached virus were removed, and fresh maintenance growth medium was added. At 24 hpi, cell and medium samples were harvested to determine the protein and mRNA levels of PEDV in Vero E6 cells, as well as the infectious virus release. (A to C) The level of viral protein and mRNA after transferrin treatment are presented in panels A and B. Quantification of the viral titer (C) was determined via plaque formation assay. (D to F) The expression of viral protein (D) and mRNA (E) after HSP70 treatment. Quantification of the viral titer (F) was determined via plaque formation assay. (G to J) Cell viability assay after transferrin, HSP70, caplain-1, and calpeptin treatment. Cell viability was determined by using a CCK-8 assay after treatment of the Vero E6 cells with different concentrations of transferrin (G), HSP70 (H), caplain-1 (I), and caplain-1 inhibitor (calpeptin) (J) for 24 h. All data are shown as means ± SD from three independent experiments. ANOVA was used to analyze differences between multiple groups, and *t* tests were used to analyze differences between two groups. *, *P < *0.05; **, *P < *0.01; ***, *P < *0.001. Download FIG S6, PDF file, 0.3 MB. Copyright © 2022 Li et al. 2022 Li et al. https://creativecommons.org/licenses/by/4.0/ This content is distributed under the terms of the Creative Commons Attribution 4.0 International license.

Subsequently, calpain-1 was purified from the protein in fraction III through affinity chromatography (Fig. 3I). Pretreatment with the purified calpain-1 reduced viral protein and mRNA expression levels, as well as the progeny virion yield, in PEDV-infected Vero E6 cells. Based on the progeny virion yield, as well as the viral mRNA expression levels, the purified calpain-1 did not show a stronger antiviral effect than that of fraction III at the same protein concentration, although it exhibited a stronger inhibition effect on intracellular viral protein expression compared to fraction III (Fig. 3J to L).

### Characterization of purified calpain-1.

The three-dimensional structure of porcine calpain-1 was predicted through homology modeling and displayed in a ribbon representation (Fig. S7A). SDS-PAGE analysis of the purified calpain-1 (Fig. S7B) revealed specific bands located around 75, 45, and 36 kDa, which were confirmed through Western blotting (Fig. S7C). Gel electrophoresis analysis confirmed that the purified calpain-1 was >75% pure. Furthermore, the secondary structure and thermal stability of the purified calpain-1 were determined through variable temperature circular dichroism (CD) analysis, revealing a high helical content (97%). As shown in Fig. S7D, the α-helical structure of the purified calpain-1 had a positive spectral band near 192 nm and a negative characteristic acromion spectral band at 208 to 222 nm. The melting temperature (*T_m_*) of the purified calpain-1 was 66°C (Fig. S7E). Moreover, the protease activity of the purified calpain-1 was confirmed (Fig. S7F).

10.1128/mbio.00358-22.7 FIG S7 Characterization of purified calpain-1. (A) Simulated three-dimensional structures of porcine calpain-1. (B) The purified porcine calpain-1 was subjected to SDS-PAGE analysis. (C) Verification of the purified protein by using Western blot analysis. (D) Circular dichroism spectra of purified calpain-1 with representative secondary structures. (E) Stability of purified calpain-1 at different temperatures, plotted as a single exponential decay curve. (F) Enzymatic activity of purified porcine calpain-1. “NC” represents a negative control, which contained the kit-provided buffer of calpain-1 and calpain-1inhibitor. “PC” represents positive control, which contained the kit-provided active calpain-1. (G) Full size gel images of calpain-1-mediated S1 protein degradation. Purified recombinant S1 protein from classical (CV777) and variant (OH851 and AJ1102) PEDV strains were incubated with calpain-1 at a ratio of 1:1 and 1:100, respectively (37°C for 2 h; 5 mM CaCl_2_ included in reaction mixture). BSA was used as a negative control. Cleavage of S1 protein by calpain-1 was determined by Western blot analysis. (H) SPR analysis of calpain-1 binding to PEDV S1 protein (CV777) or S1 (CV777) proteins lacking the b1 domain (N297 to A337). Download FIG S7, PDF file, 0.3 MB. Copyright © 2022 Li et al. 2022 Li et al. https://creativecommons.org/licenses/by/4.0/ This content is distributed under the terms of the Creative Commons Attribution 4.0 International license.

### Putative cleavage sites in PEDV spike (S) protein and their interaction with calpain-1.

**FIG 4:**
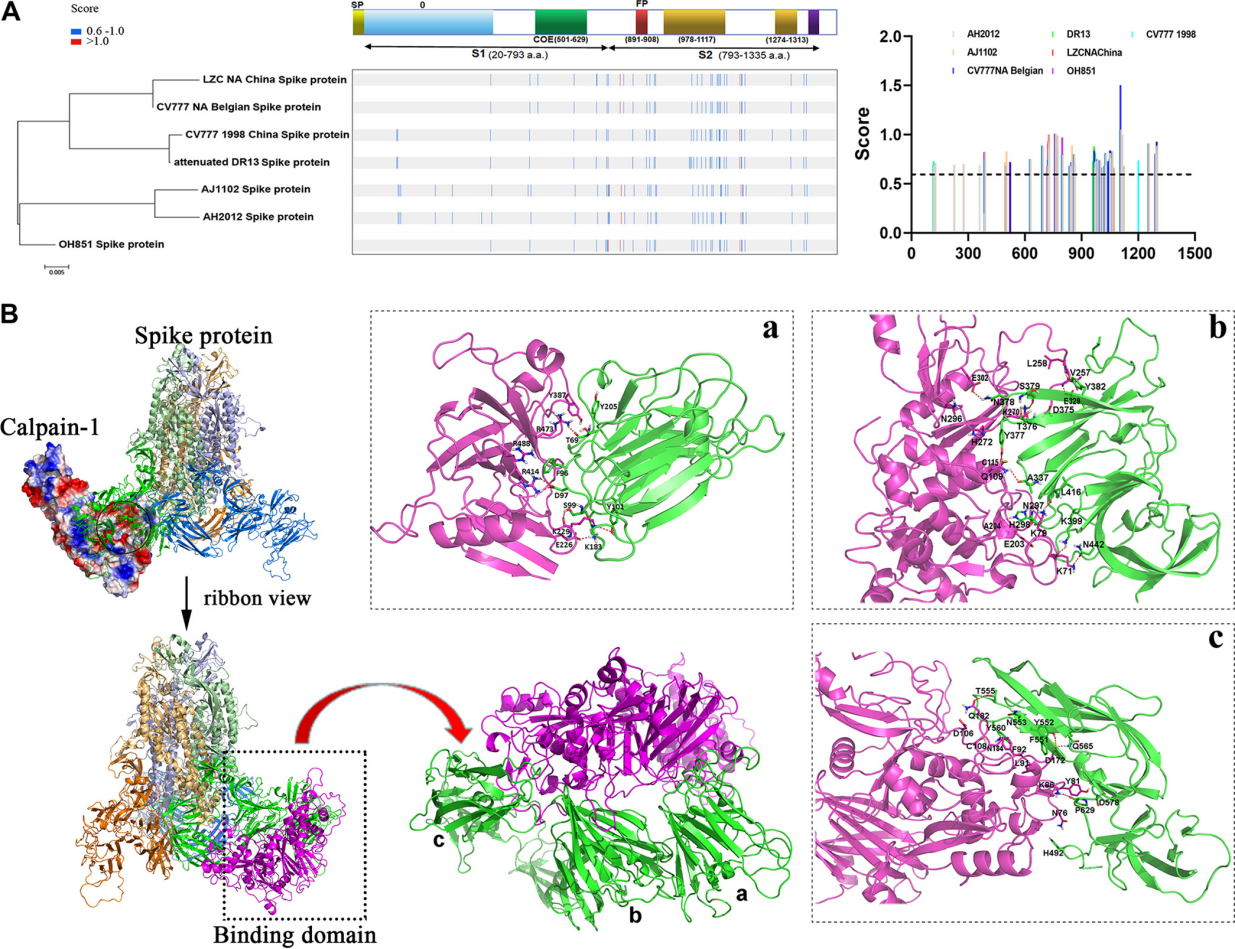
Schematic of the predicted calpain-1 proteolysis sites in PEDV spike protein and potential intermolecular interactions between molecules. (A) Bioinformatics-based prediction of putative calpain-1 cleavage sites in spike (S) protein of classical and variant PEDV strains, clustered based on their amino acid sequences. Putative cleavage scores above the maximum default cutoff value of 0.654 for different strains are presented in the bar chart. (B) Predicted bound conformation of the S protein of PEDV CV777 and calpain-1. The protein complex of calpain-1 and CV777 S protein is presented in cartoon and ribbon modes. The purple cartoon representation shows calpain-1. The S protein of CV777 is composed of three homologous subunits, which are shown as yellow, green, and blue cartoon representations. Detailed binding interactions of calpain-1 with the a, b, and c domains of S1 protein are shown in panels a, b, and c, respectively. The green cartoon representation shows S1 protein of PEDV CV777, and the purple cartoon representation shows calpain-1. Crucial residues of S1 protein and calpain-1 are presented as green and purple sticks. Hydrogen bond interactions are shown as red dotted lines.

Considering the hydrolytic characteristics of calpain-1 (31), as well as its direct inhibitory effect on viral invasion, we hypothesized that calpain-1 may influence the stability of the viral S protein, which determines and mediates viral entry into target cells. Therefore, an *in silico* approach was applied to predict calpain-1 cleavage sites within the S protein of PEDV. The identified cleavage sites (with cleavage likelihood scores above the maximum default cutoff value of 0.654) in the S protein of classical and variant PEDV strains are shown in Fig. 4A. In contrast, the S protein of the PEDV strain AJ1102 contained the highest number of putative cleavage sites (at least 41) for calpain-mediated proteolysis, which were distributed in both the S1 and S2 domains.

Subsequently, molecular docking simulations were employed to explore the nature of the interaction between calpain-1 and the S1 domain of PEDV S protein, whose structure was predicted through homologous modeling. The results indicated that the binding of calpain-1 to the S1 protein (ZDock score, 29.98) was primarily mediated through electrostatic interactions (Fig. 4B). In the a domain, Y205 of the S1 protein formed a π-π interaction with Y387 of calpain-1. T69, D97, S99, Y101, and K183 of the S1 protein formed hydrogen bonds with R473, R414, K229, and E226 of calpain-1. Additionally, F96 of the S1 protein formed a π-cation interaction with R488 of calpain-1 (Fig. 4Ba). The active site of calpain-1 mainly bound with the b domain of the S1 protein, in which Y382, E329, T376-S379, A337, L416, N297, H298, K399, and N442 of the S1 protein formed hydrogen bonds with L258, V257, E302, K270, Q109, A204, K79, and K71 of calpain-1. Additionally, Y377 of the S1 protein formed a π-π interaction with H272 of calpain-1 (Fig. 4Bb). In the c domain, T555, Y552, Q565, and P629 of the S1 protein formed hydrogen bonds with Q182, D172, and K86 of calpain-1. Moreover, H492 and D578 of the S1 protein formed polar interactions with N76 and Y81 of calpain-1 (Fig. 4Bc).

### Calpain-1 mediates proteolytic modification of PEDV S protein.

**FIG 5:**
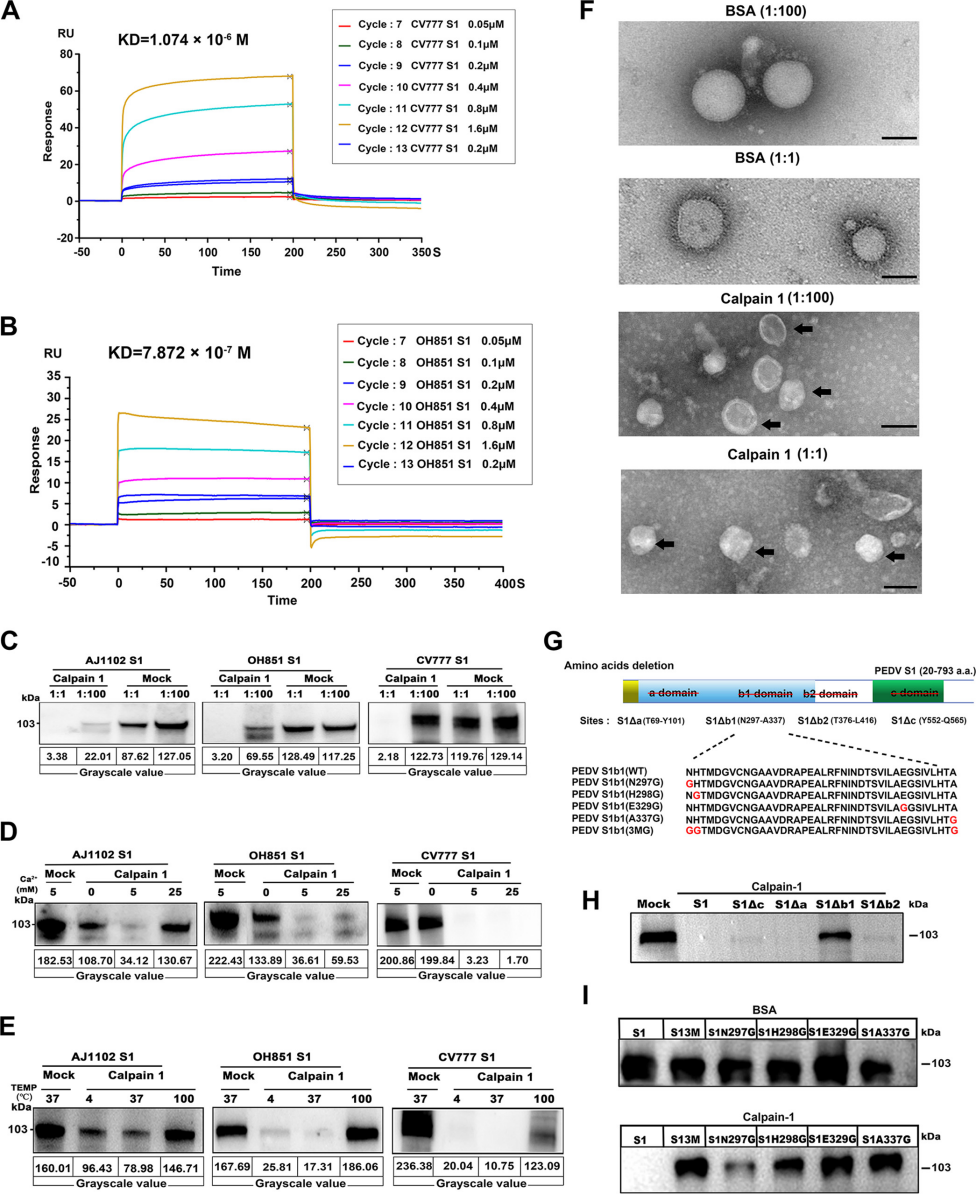
Proteolytic effects of calpain-1 on S1 domain of PEDV spike protein were analyzed *in vivo*. (A, B) Kinetic constant analysis of the interaction of calpain-1 with S1 protein of classical (CV777) PEDV (A) or variant (OH851) PEDV (B) was determined using surface plasmon resonance assay. (C) Cleavage of S1 by calpain-1 *in vitro*, as detected using Western blot analysis. Purified recombinant S1 protein from classical (CV777) and variant (OH851 and AJ1102) PEDV strains were incubated with calpain-1 at a ratio of 1:1 and 1:100, respectively (37°C for 2 h; 5 mM CaCl_2_ included in reaction mixture). BSA was used as a negative control. (D, E) Effect of calcium concentration and incubation temperature on calpain-1 activity. (D) PEDV S1 proteins were incubated with calpain-1 at a ratio of 1:1 (37°C for 2 h), while different concentrations of calcium were added to the reaction system. (E) PEDV S1 proteins were incubated with calpain-1 at a ratio of 1:1 (5 mM CaCl_2_ included in reaction mixture), while the incubation temperature was set at 4, 100, or 37°C for 2 h. (F) Effect of calpain-1 on PEDV structure visualized using transmission electron microscopy. Bar = 100 nm. Black arrows indicate viral particles with envelope-anchored S protein shedding. (G) Schematic diagram of PEDV S1 protein deletion mutants and five S1 proteins with point mutations in the b1 domain. The red line crossing out letters in the upper panel indicates the deletion of different domains (a, b1, b2 and c) in PEDV S1 protein; red letters in the bottom panel represent mutation sites. (H) Proteolytic effect of calpain-1 on deletion mutants of PEDV S1 (CV777). (I) Proteolytic effect of calpain-1 on five S1 proteins with point mutations in the b domain. BSA was used as a negative control. All data are presented as means ± SD of three independent experiments; BSA, bovine serum albumin.

Surface plasmon resonance (SPR) measurements were employed to quantify the binding between calpain-1 and the recombinant S1 proteins from the PEDV strains OH851 and CV777. The dynamic sensing diagram of real-time interaction between the S1 protein and calpain-1 is shown in Fig. 5A and B, which depicts the dynamic process of association and dissociation. SPR kinetic analyses indicated a high binding affinity between calpain-1 and the S1 protein of CV777, with a *K_D_* value of 1.074 × 10^−6^ M. The kinetic profile of the interaction between calpain-1 and the S1 protein of OH851 was similar to that between calpain-1 and the S1 protein of CV777, but the binding affinity was much higher (*K_D_* = 7.872 × 10^−7^ M).

Cleavage of the PEDV S1 protein by calpain-1 was further investigated through *in vitro* proteolysis. Calpain-1 was incubated with purified recombinant S1 proteins from classical (CV777) and variant (OH851 and AJ1102) PEDV strains, and the cleavage products were analyzed through Western blotting. The levels of all three S1 proteins significantly decreased in the presence of calpain-1, which cleaved the S1 protein in a dose-dependent manner (Fig. 5C). The full-length gels (performed in another independent experiment) were used to show the laddering effect of S1 protein degradation (Fig. S7G).

Moreover, the cleavage action of calpain-1 toward S1 protein was found to be calcium dependent between 5 and 25 mM CaCl_2_ (Fig. 5D). Calpain-1 maintained its hydrolytic activity even at low temperatures (4°C) but lost its enzymatic activity at high temperatures (100°C) (Fig. 5E). Transmission electron microscopy further confirmed that the PEDV structure was affected by the hydrolysis of the S1 protein by calpain-1 (Fig. 5F). BSA treatment did not influence the ordered arrangement of the S protein on the surface of the virions, but calpain-1 treatment resulted in significant shedding of the envelope-anchored S protein from the surface of the virus particle, particularly at the higher calpain-1 concentration.

To screen for the particular binding sites in the S1 domain of PEDV S protein required for calpain-1-mediated proteolytic modification, S1 deletion mutants were constructed according to the molecular docking results (Fig. 5G). The four mutant S1 proteins included the following: protein S1Δa lacking the a domain (T69 to Y101), protein S1Δb1 lacking the b1 domain (N297 to A337), protein S1Δb2 lacking the b2 domain (T376 to L416), and protein S1Δc lacking the c domain (Y552 to Q565). The results of comparing fragmentation of the deletion constructs after incubation with calpain-1 suggested that S1Δb1 was not efficiently hydrolyzed by calpain-1 (Fig. 5H). SPR analysis showed that lacking the b1 domain also impaired the ability of PEDV S1 to bind calpain-1. The calculated binding affinities, with a *K_D_* value of 1.985 × 10^−4^ M for protein S1Δb1 lacking the b1 domain (N297 to A337), decreased approximately 100-fold in comparison to wild-type PEDV S1 (*K_D_* = 2.504 × 10^−6^ M) (Fig. S7H). Furthermore, five mutant S1 proteins were generated with point mutations in the b1 domain (Fig. 5G), including individual mutations to glycine at N297, H298, E329, and A337 and simultaneous mutation at N297, H298, and A337 (3M). Calpain-1 incubation with the respective PEDV S1 variants indicated that the calpain-1-mediated proteolytic effect was largely abrogated by point mutations at H298, E329, and A337 of the PEDV S1 protein (Fig. 5I).

### Calpain-1 could be synthesized and produced by intestinal goblet cells.

**FIG 6:**
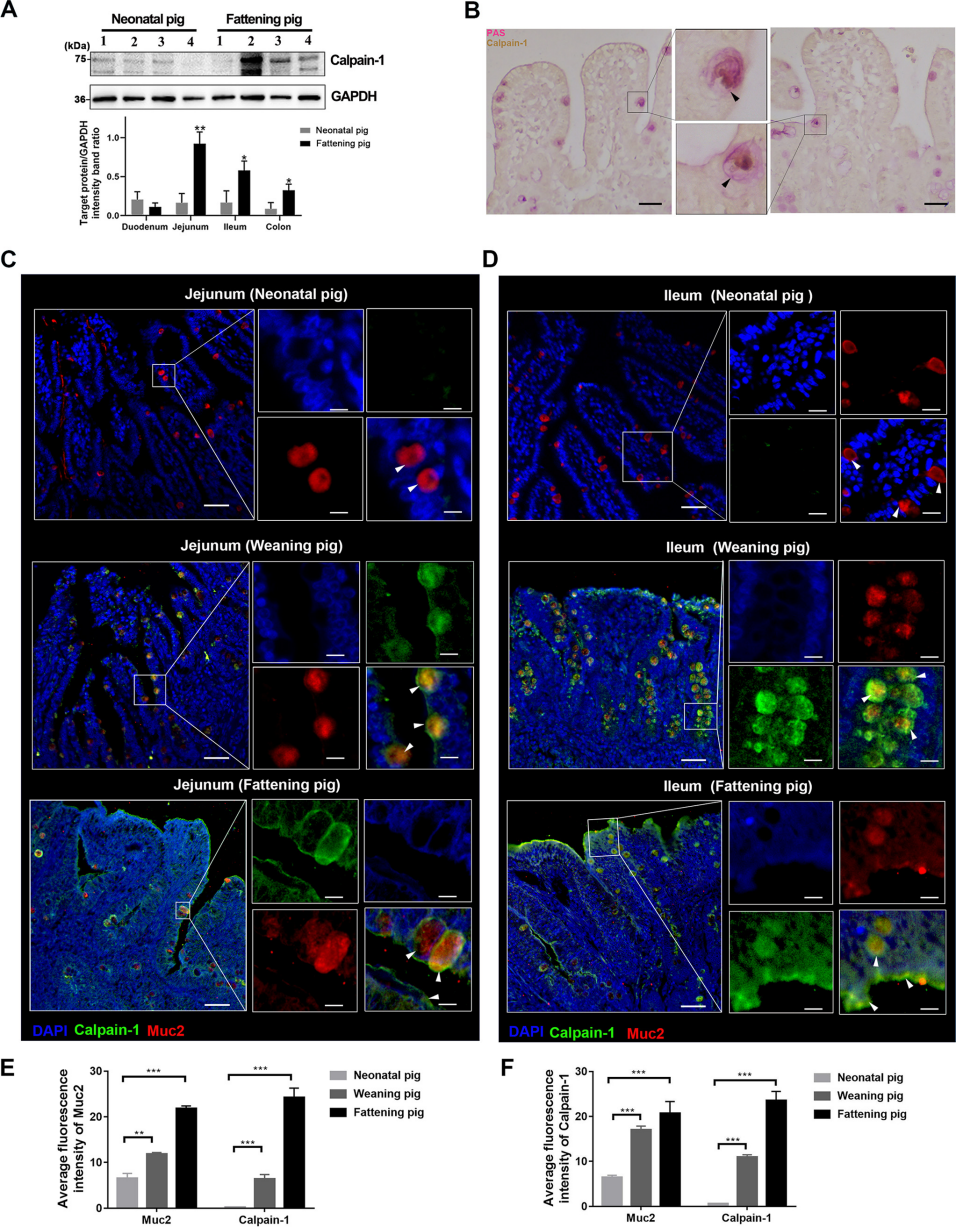
Distribution of calpain-1 in the intestines of newborn and weaned piglets. (A) Protein expression levels of calpain-1 in different intestinal segments of newborn and weaned piglets, as quantified via Western blot analysis. “1” indicates duodenum, “2” indicates jejunum, “3” indicates ileum, and “4” indicates colon. Calpain-1 expression presented in a bar chart. (B) Distribution of calpain-1 in the jejunum of weaned piglets. Calpain-1 is indicated by yellow-brown staining and marked with black arrows. (C, D) Distribution of calpain-1 in the jejunum (C) and ileum (D) of neonatal piglet, weaned piglet, and finisher pig. The nuclei were stained with DAPI (blue). Bar = 50 μm. White arrows indicate calpain-1 in the mucus layer and goblet cells. Bar = 10 μm. (E, F) Statistical results of muc2 and calpain-1 fluorescence intensity in jejunum (E) and ileum (F) of neonatal piglet, weaned piglet, and finisher pig. The ImageJ software was used for quantification of mean fluorescence intensity. The data are presented as means ± SD from three independent experiments. *, *P < *0.05; **; *P < *0.01; ***, *P < *0.001. DAPI, 4′,6-diamidino-2-phenylindole; PAS, periodic acid-Schiff; Muc2, mucin 2.

Similar to the production and secretion characteristics of the intestinal mucus, higher levels of calpain-1 were detected in the intestinal tissues of fattening piglets than newborn piglets, with the highest expression detected in the jejunum (Fig. 6A). Immunohistochemical analysis of the jejunum revealed that calpain-1 accumulated in the cytoplasm of goblet cells, which were distributed at the free surface of the jejunum villus (Fig. 6B). This observation was further confirmed through coimmunofluorescence staining of calpain-1 and Muc2. The colocalization of calpain-1 with Muc2 was detected in the jejunum and ileum mucosa of fattening and weaned piglets (Fig. 6C and D). Consistent with the Western blotting results, immunofluorescence microscopy revealed high levels of calpain-1 in the mucus layer and underlying mucosa of the intestines of fattening piglets, compared to very low levels of calpain-1 in the intestines of newborn piglets. In the fattening pigs, calpain-1 was mostly detected in the mucus layer of the intestinal epithelium; however, in weaned piglets, calpain-1 was mainly detected in goblet cells in the jejunum and ileum, particularly in the crypts of the ileum. In the crypts of the ileum, Muc2 and calpain-1 were colocalized in goblet cells, and the mucus layer was enlarged (Fig. 6C and D). Furthermore, intensity plot analysis of the immunofluorescence signals revealed the age-dependent increase of calpain-1 in the small intestinal mucosa of pigs (Fig. 6E and F).

### Oral administration of calpain-1 protects piglets against PEDV infection.

**FIG 7:**
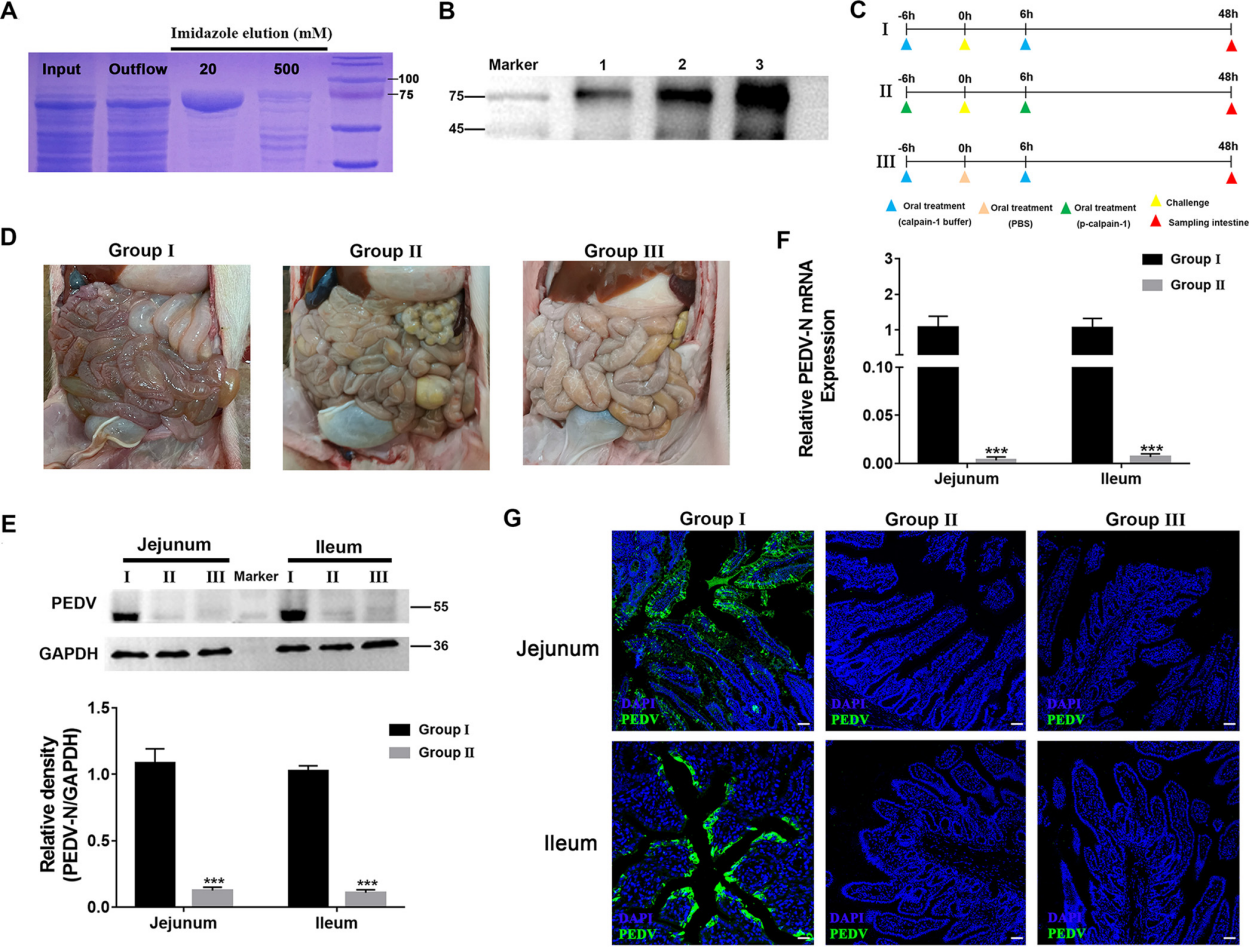
Oral administration of calpain-1 provides protection against PEDV infection. (A) SDS-PAGE analysis of the expression and purification of p-calpain-1 from 293F cells. Lane 1, total protein of 293F cells after mechanical cell disruption; lane 2, protein outflow through the Ni- nitrilotriacetic acid (NTA) column; lane 3, protein eluted with Ni buffer containing 20 mM imidazole; lane 4, proteins eluted with Ni-buffer containing 500 mM imidazole. (B) Verification of recombinant p-calpain-1 with Western blotting using anti-porcine calpain-1 MAb. Lane 1, total protein of 293F cells; lane 2, purified p-calpain-1 protein by Ni-NTA column; lane 3, calpain-1 purified from porcine mucus. (C) Schematic diagram of PEDV challenge experiment in piglets to verify the protective function of p-calpain-1. The rows represent different groups: the blank group (I), the PEDV infection group (II), and the calpain-1 treatment group (III) (2.5 mg/kg). (D) Macroscopic examination of the small intestines of piglets from each group. (E, F) Viral protein (E) and RNA (F) expression in intestinal tissues of piglets in each group (*n* = 6). (G) Distribution of PEDV in intestinal tissues of piglets from each group, as detected via immunofluorescence staining with anti-PEDV monoclonal antiboty (Mab) (green). The nuclei were stained with DAPI (blue). Bar = 50 μm. All data are presented as the means ± SD, and comparisons were performed by one-way ANOVA. PBS, phosphate buffer saline. *, *P < *0.05; **, *P < *0.01; ***, *P < *0.001.

To further verify the protective role of calpain-1 against intestinal infection in piglets, recombinant p-calpain-1 was expressed by 293F cells and purified. As shown in Fig. 7A, recombinant p-calpain-1 had an expected size of approximately 85 kDa and was expressed in a soluble form. The expression of recombinant p-calpain-1 was confirmed via Western blotting with anti-calpain-1 monoclonal antibody, and recombinant p-calpain-1 was used for subsequent trials (Fig. 7B).

Subsequently, piglets were challenged with PEDV to explore whether recombinant p-calpain-1 could protect newborn piglets against PEDV infection (Fig. 7C). At 48 hpi, only the piglets in the PEDV infection group exhibited clinical signs of infection, which included severe watery diarrhea and vomiting. The small intestines of piglets in the PEDV infection group exhibited thin and transparent intestinal walls, including an accumulation of large amounts of fluid in the intestinal lumen (Fig. 7D). In contrast, the small intestines of piglets in the calpain-1 treatment group (2.5 mg/kg) exhibited no notable pathological changes (Fig. 7D). Furthermore, PEDV viral mRNA and protein levels significantly decreased in the jejunum and ileum of piglets in the calpain-1 treatment group compared to those of piglets in the PEDV infection group (Fig. 7E and F). Immunofluorescence analysis revealed a large number of PEDV-positive cells in the jejunum and ileum of piglets in the PEDV infection group, and PEDV antigens were mainly observed in the cytoplasm of villus epithelial cells. However, PEDV antigens were undetectable in the jejunum and ileum of piglets in the calpain-1 treatment groups (Fig. 7G).

## DISCUSSION

The intestinal mucus layer has received increasing attention due to its role in intestinal protection against pathogenic infection (32). Unfortunately, research on mucus components and their protective mechanisms has long been impeded by inherent difficulties in obtaining human mucus samples. However, the gastrointestinal tissue of domestic pigs is closely related to that of humans in terms of anatomy, genetics, and physiology (33, 34), representing an ideal model for studying intestinal mucus function. The small intestinal mucus layer is composed of mucins, predominantly Muc2, secreted by goblet cells interspersed between intestinal epithelial cells. Thus, we first explored the characteristics of mucus and goblet cell distribution in porcine small intestinal tissue. A thinner mucus layer, fewer goblet cells, and decreased transcription of mucus production-related genes were observed in the small intestinal tissue of newborn piglets compared to that of weaned piglets. Considering the age-dependent resistance to PEDV, as well as a greater disease severity and higher mortality in nursing versus weaned piglets (35), an insufficient mucus barrier in the small intestine may contribute to the high susceptibility and virulence of PEDV in newborn piglets.

Furthermore, we explored the protective effect of porcine small intestinal mucus against PEDV infection. Both mucus and total mucus proteins protected epithelial cells against PEDV infection by directly blocking viral invasion. After fractioning the total mucus proteins according to molecular size and removing immunoglobulin, the retained inhibitory effect of HMWPs on PEDV infection strongly implicated the antiviral activity of Muc2. This assumption was validated by detecting the antiviral activity of Muc2 isolated from porcine small intestinal mucus. The protective role of Muc2 is mainly due to its physical properties; it forms a biopolymer matrix that binds or traps virions to block viral infection (5, 29). Therefore, the antiviral mechanism of Muc2 in the porcine small intestinal mucus was not explored in this study. As a major site of nutrient absorption, the small intestine is covered by a single layer of mucus and a loose mucin network, which is penetrable by pathogenic microorganisms (7). The protective role of the small intestinal mucus seems to depend more on defense molecules with antiviral or antibacterial activity than on the physical barrier created by Muc2 (4).

Although mucus contains a number of active molecules that are involved in intestinal protection, only a minority of these molecules have been functionally characterized (7). Therefore, our study focused on exploring novel antiviral host factors other than Muc2 in the small intestinal mucus. We found that fraction III, with a molecular weight of approximately 70 kDa, demonstrated significant antiviral activity against PEDV infection. Among the three candidate proteins in fraction III with potential protective effects against viral infection, calpain-1 exhibited substantial antiviral activity against PEDV infeciton in a dose-dependent manner, exerting a greater inhibitory effect than fraction III at equal concentrations. However, a calpain-specific inhibitor disrupted the antiviral activity of fraction III, indicating that its protective effect mainly depended on calpain-1 protease activity. Furthermore, the protective effect of calpain-1 was validated in piglets. Oral administration of calpain-1 protected PEDV-challenged piglets against PEDV infection. These findings suggest that calpain-1 may a promising antiviral component for future biomedical applications.

Calpain-1, a calcium-dependent cysteine protease found in almost all eukaryotes, is a heterodimer that consists of a catalytic subunit and regulatory subunit (CAPNS1) (36). Growing evidence suggests that calpain is associated with viral infection; in particular, its protease activity mediates the proteolytic modification of human cytomegalovirus UL112-113 proteins and enterovirus polyprotein, and calpain-1 interacts with the CD163 receptor to facilitate porcine reproductive and respiratory syndrome virus (PRRSV) uncoating in early endosomes (37, 38). Previous studies have described the role of endogenous calpain in assisting and promoting viral replication in host cells, contradicting the protective effect of secretory calpain-1 in porcine small intestinal mucus detected in our study (39, 40). We speculate that this inconsistency may be because of the following reasons. First, the function of intracellular calpain-1 might be quite different from that of secreted calpain-1 in the intestinal mucus. This phenomenon has also been reported for other proteins. For example, the functions of intracellular and extracellular phosphoglycerate kinase 1 (PGK1) differ in cancer progression. High intracellular PGK1 expression leads to tumor cell proliferation, while high extracellular PGK1 expression suppresses cancer malignancy through the suppression of angiogenesis (41). Second, the effect of calpain-1 may vary for different kinds of viruses. For example, nucleolar helicase DDX56 promotes encephalomyocarditis virus replication by interrupting interferon regulatory factor 3 phosphorylation (42), but it attenuates Chikungunya virus infection by binding and destabilizing the incoming viral genomic RNA during the replication cycle (43).

Based on the predicted cleavage site of calpain-1 in the PEDV S protein, we hypothesized that the antiviral activity of calpain-1 was likely due to its hydrolytic characteristics (44). The modeling interaction between calpain-1 and the S1 domain on the PEDV S protein, as evaluated through SPR, indicated a high binding affinity between the two molecules. *In vitro* hydrolysis experiments further revealed the calpain-1-mediated proteolysis of the S1 protein, exhibiting a clear dose- and calcium-dependent mechanism. Moreover, consistent with the distinguished calpain-1 binding capacity between S1 proteins from PEDV strains CV777 and OH851, the hydrolytic activity of calpain-1 varied between the S1 proteins of classical and variant PEDV strains. Additionally, calpain-1 maintained its hydrolytic activity at refrigerated temperatures (4°C), which significantly widens its potential applications. The construction of S1 protein mutants confirmed the critical role of the b1 domain (located between amino acids N297 and A337) in the calpain-1-mediated proteolytic effect. Considering that the active site of calpain-1 mainly binds with the b domain of the S1 protein, our results indicated that amino acid positions H298, E329, and A337 may act as potential key binding sites, forming hydrogen bonds with calpain-1 sites A204, V257, and E302, respectively. Amino acid sequence alignment revealed that the b1 domain in the S1 protein is conserved between classical and variant PEDV strains (>92.5% amino acid homology) (Fig. S8A and B), indicating the broad-spectrum activity of calpain-1 for clinical PEDV prevention.

10.1128/mbio.00358-22.8 FIG S8 Analysis of b1 domain in the S proteins of different PEDV strains. (A) Amino acid sequence alignment. (B) Homology analysis of S protein. (C and D) Doses of oral calpain-1 in newborn piglets were determined. (C) The calpain-1 concentration in intestinal mucus of newborn and weaning piglets was determined. (D) Following oral administration of various concentrations of calpain-1 for 6 h, the calpain-1 concentration in the intestinal mucus of newborn piglets was determined. As the control group, the weaning and sucking pigs were orally administered an equivalent volume of calpain-1 buffer. Download FIG S8, PDF file, 0.5 MB. Copyright © 2022 Li et al. 2022 Li et al. https://creativecommons.org/licenses/by/4.0/ This content is distributed under the terms of the Creative Commons Attribution 4.0 International license.

To date, the distribution of calpain-1 in the small intestinal mucosa has not been reported. Our study identified highly reduced calpain-1 secretion in the small intestine of newborn piglets compared with that in weaned piglets. Therefore, we hypothesize that calpain-1 deficiency in the small intestinal mucus layer may be an important factor contributing to the susceptibility of newborn piglets to PEDV infection. Additionally, the secretion of calpain-1 occurred to a significantly greater extent in the small intestine than in the large intestine, highlighting the protective role of calpain-1 against intestinal viral infection. A significant colocalization of Muc2 and calpain-1 was observed in the intestinal epithelium and lamina propria, as well as in the mucus layer, suggesting that mucosal goblet cells could synthesize and secrete calpain-1 (45). Therefore, enhanced mucin and calpain-1 production by goblet cells may prevent viral entry into the intestinal epithelium. Our future studies will focus on promoting goblet cell differentiation and enhancing their secretion function.

### Conclusion.

**FIG 8:**
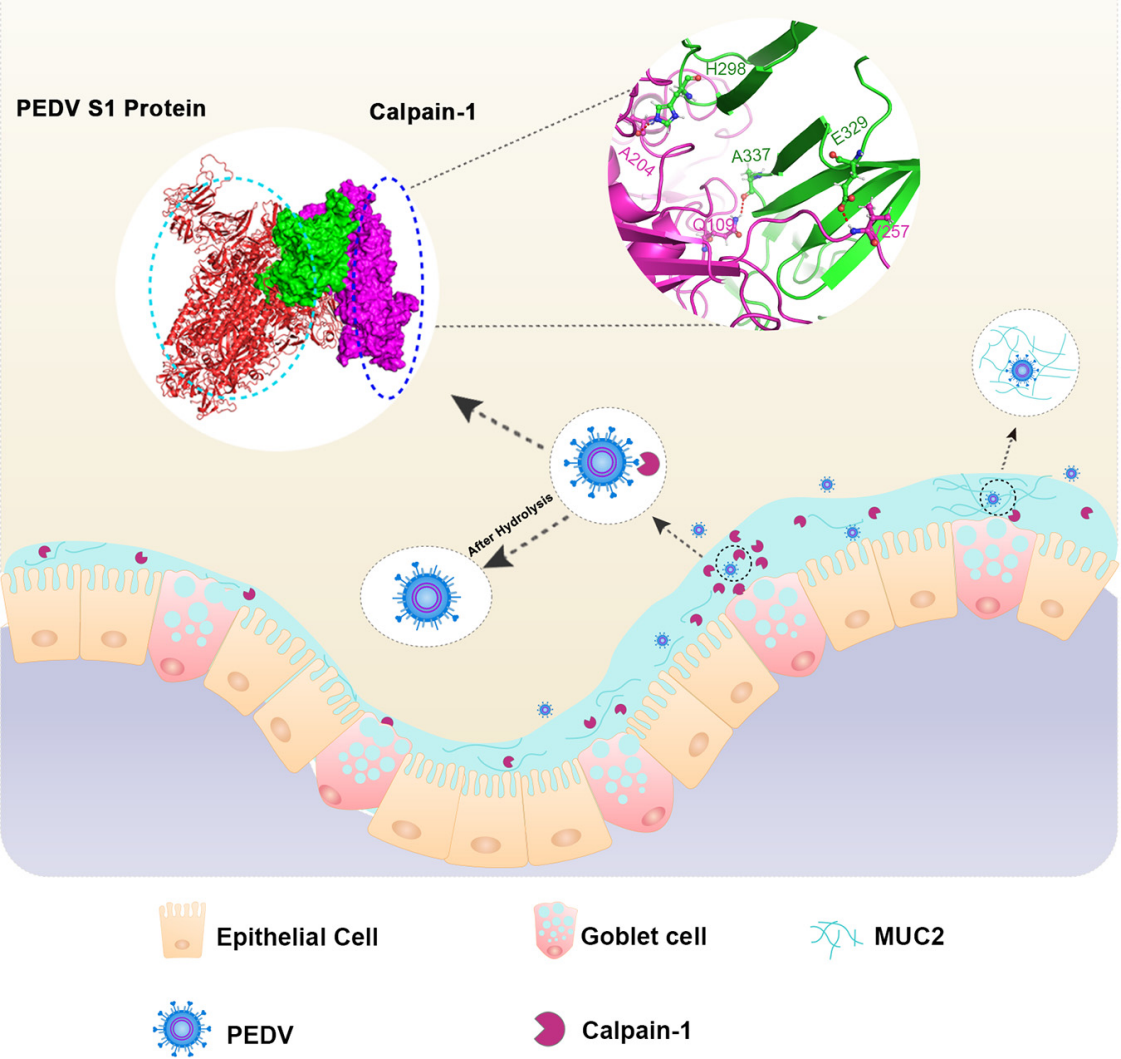
Protective mechanism of mucus protein components against intestinal PEDV infection.

In summary, our study demonstrates the protective effects of porcine small intestinal mucus against PEDV infection. The molecular mechanism underlying the antiviral activity of porcine small intestinal mucus is illustrated in Fig. 8. Among the functions of mucus protein components investigated in the current study, the antiviral effect of Muc2 against PEDV was exerted via blockade of viral diffusion and invasion through the formation of vast net-like polymers. Moreover, calpain-1 purified from porcine small intestinal mucus demonstrated potent antiviral activity both *in vitro* and *in vivo* and was mainly synthesized and produced by intestinal goblet cells. Calpain-1 directly inhibited viral invasion by binding to and hydrolyzing the S1 domain of the viral S protein. The region spanning amino acids 297 to 337 in the b domain of the PEDV S1 protein was critical for calpain-1-mediated hydrolysis, in which amino acids H298, E329, and A337 were identified as potential key binding sites. The findings of the current study improve our understanding of host factors that contribute to the protective function of the small intestinal mucus. In addition to the potential therapeutic implications for the pig industry, our findings may have implications for the prevention and control of coronavirus infection in humans.

## MATERIALS AND METHODS

### Reagents.

Porcine anti-calpain-1 polyclonal antibody (1:200, bs-1009R) was purchased from Bioss Antibodies (Shanghai, China), rabbit anti-porcine mucin-2 monoclonal antibody (Mab) (1:200, abx177613) was purchased from Abbexa Ltd. (Cambridge, UK), Muc2 polyclonal antibody (27675-1-AP) was purchased from Proteintech (Wuhan, China), and anti-PEDV N protein MAb was purchased from Medgene labs (immunofluorescence, 1:200; Western blot, 1:1,000; Brookings, SD, USA). Porcine anti-PEDV polyclonal antibody was purchased from VMRD Inc. (1:200, PC-IFA-PEDV; Pullman, WA, USA). The secondary antibodies used for immunofluorescence, such as goat anti-rabbit Alexa Fluor 488 and goat anti-mouse Alexa Fluor 594, were purchased from Invitrogen (Carlsbad, CA, USA). Goat F(ab')2 anti-rabbit IgG-(Fab')2, preadsorbed (ab102288) was purchased from Abcam (Waltham, MA, USA). Rabbit (DA1E) MAb IgG XP isotype control (no. 3900) was purchased from Cell Signaling Technology (Danvers, MA, USA). Diamidino-2-phenylindole (DAPI, 1:1,000, 2313070) was purchased from Thermo Fisher Scientific (USA). Native pig calpain-1 protein (ab198675) was purchased from Abcam (USA). Native porcine Mucin 2 (MUC2) was purchased from MyBioSource (USA). HSP70/HSPA1A protein (11660-H07H) and transferrin protein (11019-H08H) were purchased from Sino Biological (China). The calpain-1 specific inhibitor calpeptin was purchased from Selleck Chemicals (Houston, TX, USA) and was diluted to 10 mM in DMSO. Porcine calpain-1 enzyme-linked immunosorbent assay (ELISA) kits (SU-BN80625) were purchased from Ruixing Biotechnology (China). All other reagents and chemicals, unless otherwise stated, were obtained from Sigma-Aldrich (St. Louis, MO, USA).

### Cell line, viruses, and infection.

Vero E6 cells were prepared and preserved by our laboratory and cultured in Dulbecco’s modified Eagle’s medium (DMEM) containing 10% fetal bovine serum (FBS) at 37°C under 5% CO_2_. The cell line was regularly tested for mycoplasma contamination. The wild-type PEDV strain ZJ was previously obtained from the intestinal contents of a 2-day-old diarrheic piglet on a farm in Jiangsu in 2012, and the strain was clustered with the emerging virulent strain based on phylogenetic analysis (46). Briefly, a confluent monolayer of Vero E6 cells was inoculated with PEDV at a multiplicity of infection (MOI) of 0.1 for 1 h at 37°C. The inoculum and unattached virus were removed by washing with DMEM, maintenance medium (DMEM with 2% FBS) was added, and the culture was incubated at 37°C under 5% CO_2_. The infected cells were analyzed after the required incubation period. To harvest the virus, the infected cells were subjected to one freeze-thaw cycle with a cytopathic effect (CPE) of 80%, and the cell supernatant was harvested for further propagation or stored at −80°C.

### Viral titer.

The viral titer of the cell supernatant was measured through plaque assay. The confluent monolayers of Vero cells that were cultivated in six-well tissue culture plates were infected with 500-μL aliquots of serial 10-fold dilutions of the collected supernatant. After incubation for 1 h at 37°C, the cells were overlaid with 1% agarose in DMEM and incubated for 3 days at 37°C. Subsequently, plaques were visualized by staining with crystal violet and counted.

### Animals.

The thickness of mucus layer, number and functional gene expression of goblet cells, and calpain-1 expression and distribution were quantified in the intestines of weaned (aged 1 month), fattening (aged 2 months), and newborn piglets (aged 1 day and 7 days). As the amount of small intestinal mucus from weaned piglets could not meet the needs of our experiment. The mucus used in our study was isolated from the small intestine of a finisher pig at 260 days of age. Each time, the mucus was isolated from three different pigs (healthy littermates), with three technical replicates of mucus samples per pig. All piglets used in the study were obtained from a swine herd at the Jiangsu Academy of Agricultural Science and were artificially fed with milk. The swine herd was seronegative for antibodies against PEDV, porcine reproductive and respiratory syndrome virus (PRRSV), porcine respiratory coronavirus (PRCV), transmissible gastroenteritis virus (TGEV), and porcine circovirus type 2 (PCV2). All animal procedures and experiments were performed according to protocols approved by the Institutional Animal Care and Use Committee of Nanjing Agricultural University (Nanjing, China) and followed the National Institutes of Health guidelines (approval PT20200523005).

### Measurement of mucus thickness.

Three newborn and weaned piglets were anesthetized with pentobarbital sodium (20 mg/kg body weight), and a midline incision was made slightly anterior to the navel. Intestinal segments (3 cm in length) were injected with DyLight 594-labeled latex beads (Sigma, USA, L3280). During the procedure, the piglets were kept warm on a 37°C warming pad. After 1 h, the intestines were removed, embedded in OCT tissue freezing medium (Sakura, Torrance, CA), and cut into 8-μm sections for immunofluorescence staining, as described below. The sections were sealed with 10% glycerin and observed via CLSM (LSM 710; Zeiss, Wetzlar, Germany) at an excitation wavelength of 594 nm.

### Mucus preparation and total protein extraction.

The small intestinal mucus was aspirated and purified as previously described (14). Briefly, mucus was scraped from the small intestine of the pig, solubilized in phosphate-buffered saline (PBS) supplemented with protease inhibitor cocktail (1:100 dilutions; Sigma-Aldrich) and antibiotics (2% penicillin/streptomycin and 100 μg/mL gentamicin; Gibco, Waltham, MA, USA). The insoluble fraction of the crude mucus was removed via centrifugation at 5,000 rpm for 5 min at 4°C. The total mucus proteins were obtained by means of ammonium sulfate precipitation (45% final concentration of ammonium sulfate), as previously described (47).

### PAGE and electroelution of mucus protein.

The LMWPs in the small intestinal mucus were subjected to 10% native PAGE to separate proteins with biological activity. Native gel sample loading buffer (4×) was added to the total mucus proteins, and separation was performed in electrophoretic buffer (Tris-glycine) on ice at 4°C and 70 V/gel for 4.5 h. The gel was placed in sample buffer without mercaptoethanol, SDS, or heat and stained with 4 mg/mL Coomassie brilliant blue R-250. Recovery of mucus LMWPs from fractions I-IV was performed using the model 422 Electro-Eluter (Bio-Rad Laboratories, Hercules, CA, USA), according to the manufacturer’s instructions. The protein concentration of each collected fraction was estimated using the Micro BCA protein assay kit (Pierce Biotechnology, Waltham, MA, USA), according to the manufacturer’s instructions. Fractions were stored at −20°C in 50% vol/vol glycerinum.

### Mucus protein separation using size-exclusion chromatography.

The fraction III protein in mucus LMWPs were separated by size-exclusion chromatography using a Zenix SEC-150 gel-filtration column (3 μm, 150 Å, 7.8 × 300 mm) with an AKTA purifier system (GE Healthcare, Chalfont Saint Giles, UK). The sample was processed at a flow rate of 0.8 mL/min in standard PB buffer (150 mM, pH 7.0) containing protease inhibitor cocktail. Eluted proteins were collected and maintained at 4°C.

### Liquid chromatography with tandem mass spectrometry (LC-MS/MS) analysis.

The purified fraction III from LMWPs was lyophilized, desalted using a StageTip (Thermo Fisher Scientific, Waltham, MA, USA), and analyzed by LC-MS/MS using a Q-Exactive mass spectrometer equipped with an EASY nLC1000 nano-flow ultra-high-pressure liquid chromatography system (Thermo Fisher Scientific). Gradient elution was performed using mobile phases A (0.1% formic acid in water) and B (0.1% formic acid in acetonitrile). The flow rate was set at 0.3 mL/min. Electrospray ionization (ESI) was performed in positive ion mode with a mass range from 350 to 1,800 *m*/*z*. After optimization, the source parameters were set as follows: nebulizer ion spray voltage, 1.8 kV; and ion transport capillary temperature, 250°C. Data were acquired and processed using Proteome Discoverer 2.3 software (Thermo Fisher Scientific) and the UniProt-SUS database.

### Purification of calpain-1.

Purification of calpain-1 was performed through Ni-nitrilotriacetic acid (NTA) affinity chromatography. The antibody for porcine calpain-1 was immobilized via amine coupling to a 1-mL HiTrap NHS-activated HP column (GE Healthcare) following the manufacturer’s protocol. The column was equilibrated with two column volumes of equilibration buffer (20 mM NaH_2_PO_4_, 20 mM Na_2_HPO_4_, and 0.5 M NaCl, pH 7.4) prior to the affinity purification step. Then, fraction III was loaded onto the calpain-1 antibody-coated affinity column, and unbound proteins were removed by washing four times with a binding buffer (20 mM NaH_2_PO_4_, 20 mM Na_2_HPO_4_, and 2 M NaCl, pH 7.4). Bound proteins were eluted from the column with a glycine-based elution buffer (pH 2) and dialyzed in Tris buffer (pH 8.0). The eluted proteins were analyzed by SDS-PAGE and Western blotting.

### Cell viability assay.

The activity of Vero E6 cells was assayed using the CCK-8 (Yeasen, 40203ES60) method, according to the manufacturer’s instructions. Vero E6 cells were seeded at 5 × 10^4^ cells/well in 96-well plates and incubated for 12 h. Different amounts of small intestinal mucus, Muc2, protein fraction III, calpain-1, HSP70, and transferrin were added to the culture medium, and the cells were treated for the indicated times, followed by washing and incubation with fresh medium for 24 h (37°C). After adding CCK-8 reagent and incubating for 4 h, the optical density (OD) value was measured at 570 nm using a microplate reader.

### Plaque reduction assay.

Vero E6 cells were seeded at 2 × 10^5^/well in 12-well cell culture plates and incubated for 12 to 18 h at 37°C until reaching approximately 95% confluence. PEDV (10^3^ PFU) was individually mixed with small intestinal mucus, native porcine calpain-1, electrophoretic HMWPs, LMWP fractions I to IV, or BSA (negative control), and the mixtures were incubated at 37°C for 1 h. The protein mixtures were added to the cells and incubated for 30 min at 4°C to allow virus adsorption, followed by incubation at 37°C for 30 min. Subsequently, the cell supernatant was removed, the cells were washed to remove any unattached virus, and DMEM containing 1% agar was added to each well. After incubation for 72 h at 37°C, the cells were fixed with 4% formaldehyde, the agar overlay was removed, and the plaques were visualized by staining with 0.1% crystal violet and counted.

### Quantitative reverse-transcription PCR (RT-qPCR).

Total RNA from different tissues and cells was extracted using TRIzol reagent (Invitrogen) according to the manufacturer’s instructions. Reverse transcription was completed using the PrimeScript RT reagent kit (TaKaRa Bio, Beijing, China). RT-qPCR was performed using TB Green Premix EX Taq II (TaKaRa Bio) with the 7500 Fast real-time PCR system (Applied Biosystems, Foster City, CA, USA). Cellular GAPDH was used as the internal control to quantify cDNA amounts. Relative expression levels of RNA were calculated using the 2^−ΔΔCT^ method. All primers used for RT-qPCR analysis are listed in Table 1.

**TABLE 1:** Primer sequences used for reverse transcription-quantitative PCR

Genes	Primers	Sequence (5′ to 3′)
PEDV (N)	Forward	CACCTCCTGCTTCACGTACA
	Reverse	AGCTCCACGACCCTGGTTAT
GAPDH (Vero)	Forward	ACATCATCCCTGCCTCTACTG
	Reverse	CCTGCTTCACCACCTTCTTG
Muc1 (Sus scrofa)	Forward	ACACCCATGGGCGCTATGT
	Reverse	GCCTGCAGAAACCTGCTCAT
GAPDH (Sus scrofa)	Forward	GATGCCCTGGCCACAGAA
	Reverse	ACCCCTGCTCCCTCAACATC
Muc2 (Sus scrofa)	Forward	GGGGTCCCCGTCTTCTTCAA
	Reverse	GCGGTCCAGTCTGCTGTGTTG
Muc4 (Sus scrofa)	Forward	GATGCCCTGGCCACAGAA
	Reverse	TGATTCAAGGTAGCATTCATTTGC
Tff1 (Sus scrofa)	Forward	CTGCTTCGACTCCAGCATC
	Reverse	CAGAAGGTGCATTCTGTTTCC

### Antibody blocking assay.

Prior to the assays determining the antiviral activity of purified HMWPs, the protein mixture was pretreated with blocking antibodies against Muc2 (27675-1-AP) with a certain concentration gradient (1, 2, and 10 μg/mL). Then, the viral titers in the supernatant, as well as the production of viral RNA in epithelial cells from different treated groups, were detected.

### Bioinformatic analysis.

Bioinformatics-based computational analysis was utilized to predict protein structures. The following programs were employed to identify cleavage sites in calpain substrates: CaMPDB (http://calpain.org), LabCaS (http://www.csbio.sjtu.edu.cn/bioinf/LabCaS), and GPS-CCD (http://ccd.biocuckoo.org).

### *In silico* docking.

The crystal structure of the PEDV S protein was obtained from the Protein Data Bank (UniProtKB no. Q91AV1, PDB code 6U7K). However, the S1 domain of the PEDV S protein had multiple deletions in sequence 26 to 734, including sections 26 to 42, 56 to 72, 80 to 160, 185 to 199, 218 to 220, and 355 to 362. The missing sections other than 26 to 42 and 80 to 160 were completed using the Build Loops tool in BIOVIA Discovery Studio version 4.1 software, and section 80 to 160 was completed using another SPIKE_PEDV7 structure (PDB code 6VV5) to complete the template model and then obtain the three-dimensional structure of the SPIKE_PEDV7 sequence for sequence 43 to 1319. A BLAST search of Sus scrofa calpain-1 against the PDB database identified rat calpain-1 (PDB code 1QXP) with the highest homology (up to 81.9%). After downloading the structure of rat calpain-1 from the database, the B chain was deleted, and the Prepare Protein tool in Discovery Studio was adopted to construct the missing loops. The Align Sequence tool in the Templates module was used to perform sequence alignment between S. scrofa calpain-1 and the prepared rat calpain-1, and modeling was conducted using the Build Homology Models tool. The model with the highest score was selected for optimization to obtain the structure of S. scrofa calpain-1. The proton and energy of the protein structure were optimized using the CHARMM force field in the Prepare Protein module. The obtained results were used for subsequent molecular docking.

The Dock Proteins tool (ZDock) in Discovery Studio was used to model molecular docking of calpain-1 to the PEDV S protein. The angular step size was set to 6 Å. A total of 2,000 docking conformations were generated, scored by ZRank, and clustered into 100 classes. The root mean square deviation (RMSD) cutoff and interface cutoff were set to 10.0. The obtained 2,000 conformations were screened using the Process Poses (ZDock) module of Discovery Studio, preserving conformations in which interactions occurred between the active site region of calpain-1 and the S1 domain of the PEDV S protein. The conformation with the highest ZDock score was selected for energy optimization, and the interaction mode was analyzed.

### Surface plasmon resonance (SPR).

SPR experiments were performed on a Biacore ATC-018 S200 instrument (GE Healthcare) at room temperature. Calpain-1 was immobilized at a coupling density of 8,860 resonance units (RU) on a CM5 sensor chip using the amino-coupling method. HEPES buffer (26.25 mM HEPES, 105 mM NaCl, 3.15 mM dithiothreitol (DTT), and 5.25 mM CaCl_2_) was used as the immobilization and binding solution. One channel (Fc2) was used for the immobilized calpain-1, while the other (Fc1) was used as a reference surface for nonspecific binding measurements.

The binding of the immobilized calpain-1 to the PEDV S1 protein was further explored. The recombinant S1 proteins of PEDV strains CV777 and OH851 were obtained via prokaryotic expression systems. All SPR experiments were performed in the running buffer (pH 5.4) containing 25 mM HEPES, 100 mM NaCl, and 3 mM EDTA supplemented with 5 mM CaCl_2_. To generate binding data, PEDV S1 protein at concentrations ranging from 100 to 0.8 μM was injected over immobilized calpain-1 at a constant flow rate of 90 mL/min for 200 s. S1 protein dissociation was monitored by flowing running buffer at 90 mL/min for 200 s. The data were collected, and kinetic parameters were determined using Biacore T200 evaluation software (GE Healthcare). The binding data were fit using the 1:1 Langmuir binding model to obtain individual *K*_on_ and *K*_off_ kinetic constants, and individual values were combined to derive average *K_D_* values. The experiments were conducted in triplicate with fresh immobilization. All reagents were purchased from GE Healthcare (Uppsala, Sweden).

### Proteolytic cleavage assay.

Purified recombinant PEDV S1 protein (100 μg/mL) was mixed with porcine calpain-1 or BSA in 1:1 or 1:100 ratios with buffer containing 25 mM HEPES (pH 7.0), 100 mM NaCl, 3 mM DTT, and varied concentrations of CaCl_2_ (0, 5, and 25 mM). After incubation at 37 or 4°C for 1 h, the reaction was terminated by adding 10 mM EDTA. As a negative control, calpain-1 was heat-inactivated at 95°C for 15 min (boiled).

### Enzyme activity assay.

The enzymatic activity of calpain-1 was measured using a calpain activity fluorometric assay kit (Biovision, Mountain View, CA, USA), as per the manufacturer’s instructions. As a positive control, 1 μL of active calpain was added to 85 μL buffer. As a negative control, calpain inhibitor (1 μL) were added to the buffer. To each assay, 10 mL of 10× reaction buffer and 5 μL of calpain substrate were added and incubated at 37°C for 1 h in the dark. The samples were measured using a microplate reader equipped with a 400-nm excitation filter and a 505-nm emission filter.

### Variable temperature circular dichroism (CD) spectroscopy.

The secondary structure and melting temperature (*T_m_*) of calpain-1 were examined through CD spectroscopy using a J715 spectropolarimeter (JASCO Inc., Easton, MD, USA) in the far-UV range with a 0.1-cm-width cuvette. The ellipticity of samples was measured in the presence of 8 mol/L urea. For sample preparation, 300 μL protein solution was mixed with 200 μL 8 mol/L urea solution in an ultrafiltration tube. The sample was centrifuged twice at 12,000 relative centrifugal force (rcf) for 10 min at 4°C, 200 μL double-distilled H_2_O was added, and the sample was centrifuged again under the same conditions. Sample testing was performed with a starting temperature of 20°C and an ending temperature of 95°C. CD measurements were acquired at a step size of 1 nm with 1°C/point. The spectrum of the background (buffer only) was measured and subtracted from the sample spectrum.

### Cloning and expression of p-calpain-1.

Full-length porcine calpain-1 was custom synthesized by Sangon Biotech (Shanghai, China) and inserted into the eukaryotic expression vector pcDNA3.4. Subsequently, the calpain-1 expression plasmid (pcDNA3.4-p-calpain-1) was extracted and purified using the EndoFree plasmid maxi kit (Qiagen, Hilden, Germany) from transformed Escherichia coli, according to the manufacturer’s instructions. The pcDNA3.4-p-calpain-1 plasmid was then transfected into HEK 293F cells using Lipofectamine 2000 (Invitrogen). After 48 h, recombinant protein (p-calpain-1) expressed in cells was analyzed through Western blotting. Finally, the cells were broken by sonication, the extract was clarified by centrifugation, and p-calpain-1 was purified using Ni-NTA column chromatography.

### The optimal concentration for *in vivo* calpain-1 applications was determined.

The findings above suggested that newborn piglets had significantly lower calpain-1 secretion in their small intestine than weaned pigs. PEDV infection shows age-dependent pathogenicity by causing serious enteric disease in newborn piglets and mild disease or asymptomatic infections in weaning or older pigs (48, 49). Calpain-1 deficiency in the small intestinal mucus layer could be an important factor contributing to the susceptibility of newborn piglets to PEDV infection. Therefore, we believe that exogenous supplementation to achieve the same physiological concentration of calpain-1 in the small intestinal mucus of newborn piglets as that in weaned piglets could effectively protect newborn piglets from PEDV infection.

Based on these observations, a series of animal experiments were carried out to determine the optimal concentration of calpain-1 for *in vivo* applications. First, 5 cm of jejunum were collected from newborn and weaned piglets. Mucus was scraped from each intestinal segment, and total mucus proteins were extracted. The calpain-1 concentrations in mucus were determined by ELISA and ranged from 0.73 to 1.56 μg/μL in newborn piglets and 3.97 to 6.13 μg/μL in weaned piglets (Fig. S8C). Newborn piglets were orally administered different concentrations of calpain-1, and the calpain-1 concentration in the mucus of the jejunum was detected 6 h after oral administration. The concentrations of calpain-1 in the intestinal mucus of newborn piglets were the same as those in weaned piglets following the oral intake of 2.5 mg/kg recombinant calpain-1 (Fig. S8D). Therefore, 2.5 mg/kg calpain-1 was orally administered to further validate the protective effect of calpain-1 in the PEDV challenge experiments.

### PEDV challenge experiments.

Nine newborn piglets with similar weights were allocated to three groups and housed in three separate rooms for 24 h prior to the experiment. The three groups were as follows: the PEDV infection group (I), the calpain-1 treatment group (II), and the blank group (III) as negative control. Piglets in group II were orally administered 2.5 mg/kg calpain-1. Piglets in groups I and III were orally administered the same amount of calpain-1 buffer containing 25 mM HEPES (pH 7.0), 100 mM NaCl, 3 mM DTT, and 5 mM CaCl_2_. After 6 h, piglets in groups I and II were challenged with 1 mL PEDV (10^4^ PFU/mL) administered by oral inoculation, while piglets in group III received the same volume of DMEM. At 6 h post-PEDV infection, the piglets in group II were treated with calpain-1 (2.5 mg/kg) again, and the piglets in other groups were orally administered the same amount of calpain-1 buffer. The animals were artificially fed with milk every 3 h throughout the experiment to meet or exceed the nutritional requirements for piglets (NRC, 2012). After PEDV challenge, the piglets were observed daily for symptoms of diarrhea. Severe watery diarrhea with vomiting was observed in piglets in group I at 48 h postinfection. Piglets were anesthetized with 100 mg/kg pentobarbital sodium and sacrificed for macroscopic examination, and tissues were collected.

### Immunofluorescence staining.

Paraffin-embedded intestinal tissue sections were dewaxed, and antigens were retrieved using antigen repair solution (pH 9.0) containing EDTA. Sections were permeabilized with 0.4% Triton X-100, followed by blocking with 5% BSA in PBS for 1 h. After blocking, the sections were incubated with rabbit polyclonal anti-Muc2 (1:100) for 12 h at 4°C, followed by DyLight 594-labeled goat anti-rabbit IgG (1:200) at 25°C for 1 h. The sections were then incubated with rabbit IgG (Cell Signaling Technology) for 1 h to saturate open binding sites on the first secondary antibody, thereby preventing binding with the second primary antibody. Moreover, the rabbit IgG was blocked using goat anti-rabbit IgG-(Fab')2. Finally, the sections were stained with anti-calpain-1 antibody (1:100) followed by DyLight 488-conjugated goat anti-rabbit IgG (1:200). All incubations were performed in a wet box to prevent sections from drying. The sections were visualized by CLSM using an LSM 710 immunofluorescence microscope (Zeiss). Images were analyzed using ZEN 2012 software (Zeiss). Fluorescence quantification was performed using ImageJ software.

### Western blot analysis.

Homogenized tissue or cells were lysed in ice-cold RIPA buffer containing a protease inhibitor cocktail (Thermo Fisher Scientific). Protein samples were assessed through Western blotting using specific primary antibodies. Proteins were quantified using ImageJ software. Band intensity was measured and normalized against GAPDH expression. Uncropped Western blot images can be found in the Fig. S9. All Western blots were performed in triplicate.

10.1128/mbio.00358-22.9 FIG S9 Triplicates of original Western images for all relevant Western blots results. Shown are the original blot images for Fig. 3L, Fig. 5C, Fig. 5D, Fig. 5E, Fig. 5H, Fig. 5I, Fig. 6A, Fig. 7B, Fig. 7E, Fig. S3C, Fig. S4A, Fig. S4C, Fig. S5C, Fig. S5D, Fig. S6A, and Fig. S6D. Download FIG S9, PDF file, 1.4 MB. Copyright © 2022 Li et al. 2022 Li et al. https://creativecommons.org/licenses/by/4.0/ This content is distributed under the terms of the Creative Commons Attribution 4.0 International license.

### Statistical analysis.

The results are expressed as means ± standard deviation (SD) and were analyzed using SPSS version 17.0 software (SPSS Inc., Chicago, IL, USA). One-way analysis of variance (ANOVA) was employed to determine significant differences among multiple groups, and *t* tests were employed to determine differences between two groups. Significance was set as follows: *, *P < *0.05; **, *P < *0.01; and ***, *P < *0.001. The data were combined from at least three independent experiments unless otherwise stated.
