# Supplementary material for: Calpain-1: a Novel Antiviral Host Factor Identified in Porcine Small Intestinal Mucus
Source: mBio. 2022 Sep 14;13(5):e00358-22. doi: 10.1128/mbio.00358-22 (PMC9600339; doi:10.1128/mbio.00358-22)
Supplement: FIG S1 [file mbio.00358-22-s0001.pdf]

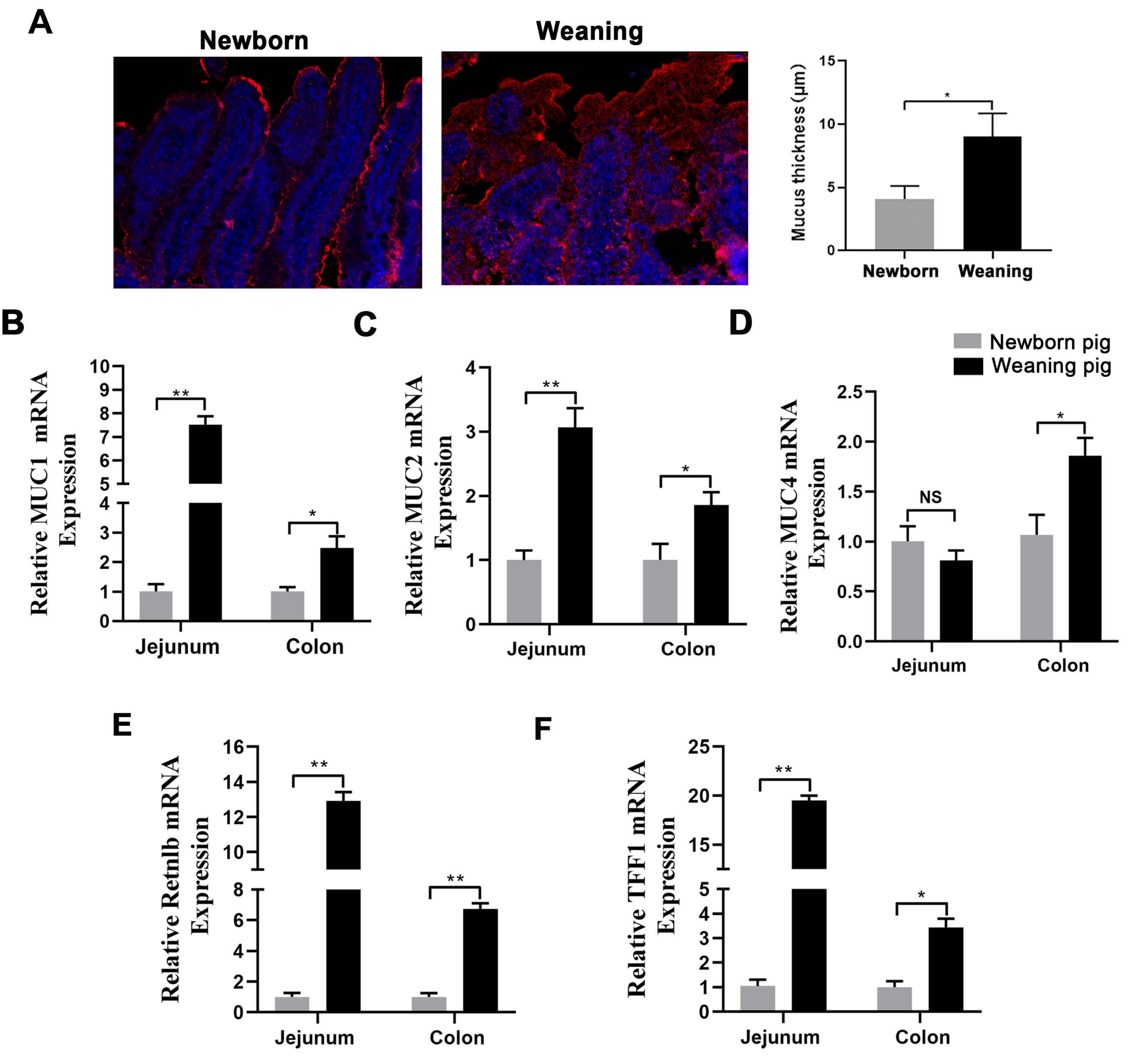

A
Newborn
Weaning
Mucus thickness (μm)
15
10
5
0
*
Newborn
Weaning
B
Relative MUC1 mRNA Expression
10
9
8
7
6
5
4
3
2
1
0
**
*
Jejunum
Colon
C
Relative MUC2 mRNA Expression
4
3
2
1
0
**
*
Jejunum
Colon
D
Newborn pig
Weaning pig
Relative MUC4 mRNA Expression
2.5
2.0
1.5
1.0
0.5
0.0
NS
*
Jejunum
Colon
E
Relative Retnlb mRNA Expression
16
14
12
10
8
6
4
2
0
**
**
Jejunum
Colon
F
Relative TFF1 mRNA Expression
25
20
15
5
4
3
2
1
0
**
*
Jejunum
Colon
