# Supplementary material for: Calpain-1: a Novel Antiviral Host Factor Identified in Porcine Small Intestinal Mucus
Source: mBio. 2022 Sep 14;13(5):e00358-22. doi: 10.1128/mbio.00358-22 (PMC9600339; doi:10.1128/mbio.00358-22)
Supplement: FIG S2 [file mbio.00358-22-s0002.pdf]

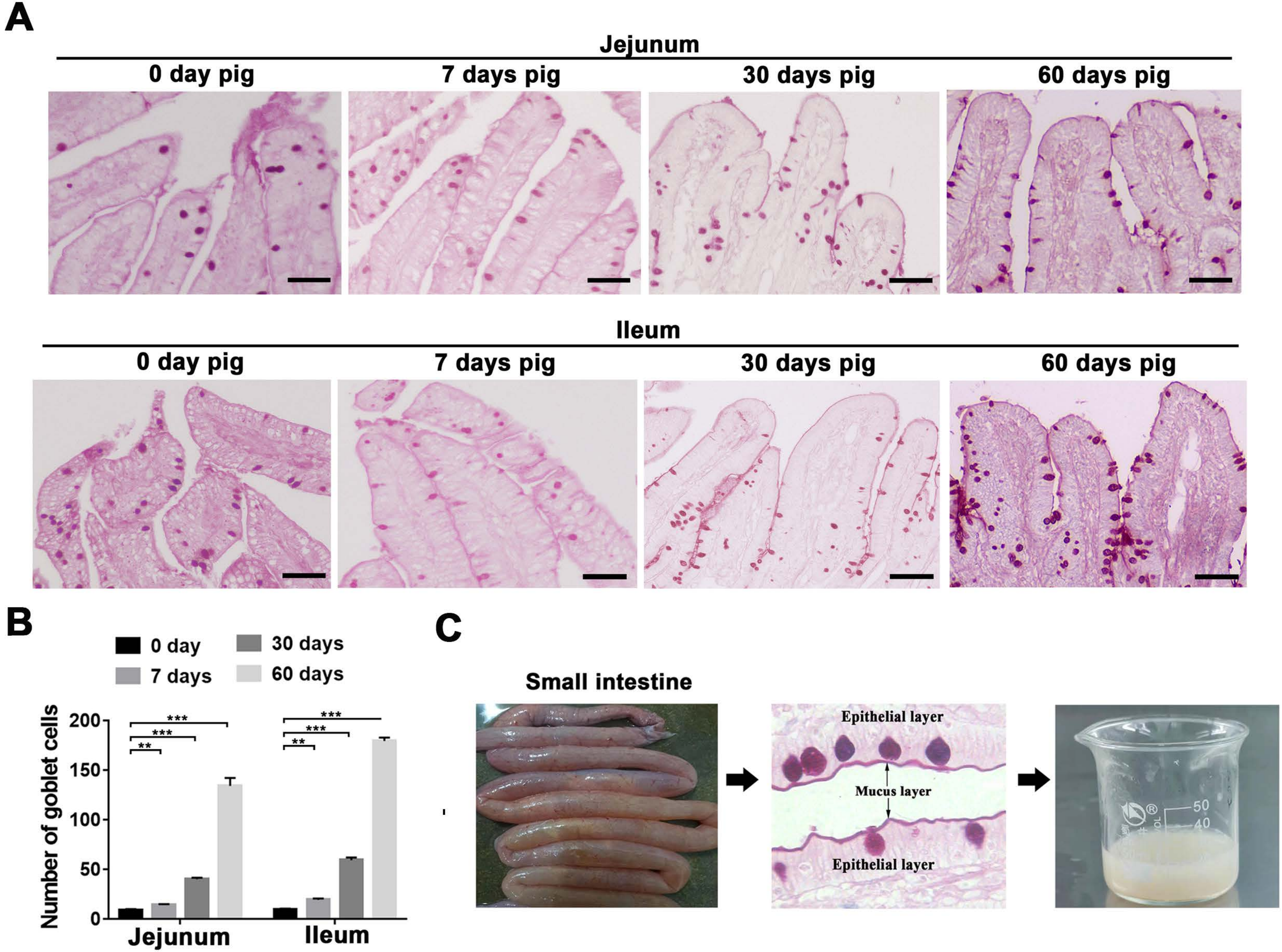
A
Jejunum
0 day pig
7 days pig
30 days pig
60 days pig
Ileum
0 day pig
7 days pig
30 days pig
60 days pig
B
0 day
7 days
30 days
60 days
Number of goblet cells
200
150
100
50
0
**
***
***
Jejunum
Ileum
C
Small intestine
Epithelial layer
Mucus layer
Epithelial layer
50
40
