# Supplementary figures and images for: Calpain-1: a Novel Antiviral Host Factor Identified in Porcine Small Intestinal Mucus
Source: mBio. 2022 Sep 14;13(5):e00358-22. doi: 10.1128/mbio.00358-22 (PMC9600339; doi:10.1128/mbio.00358-22)

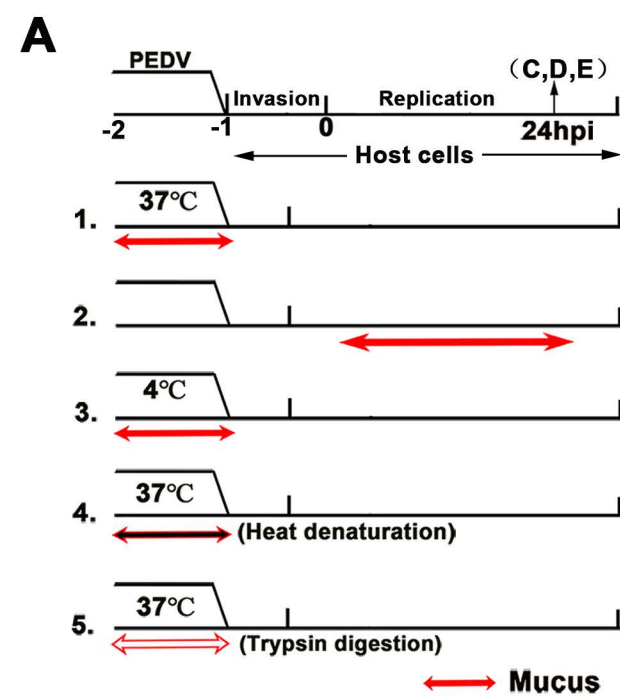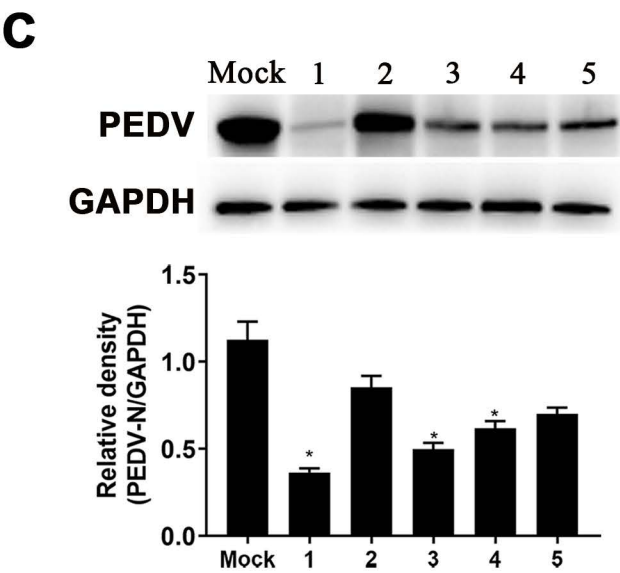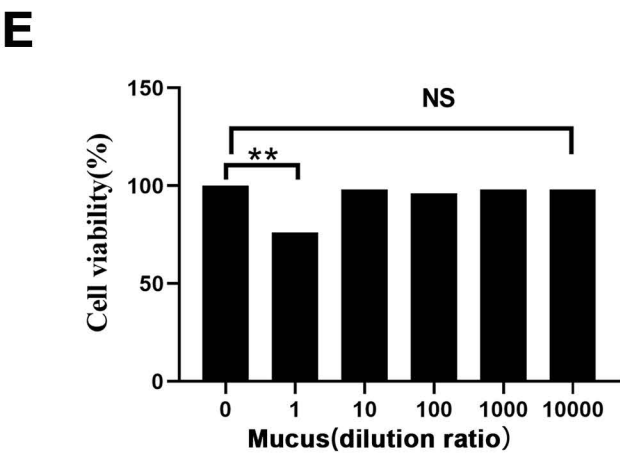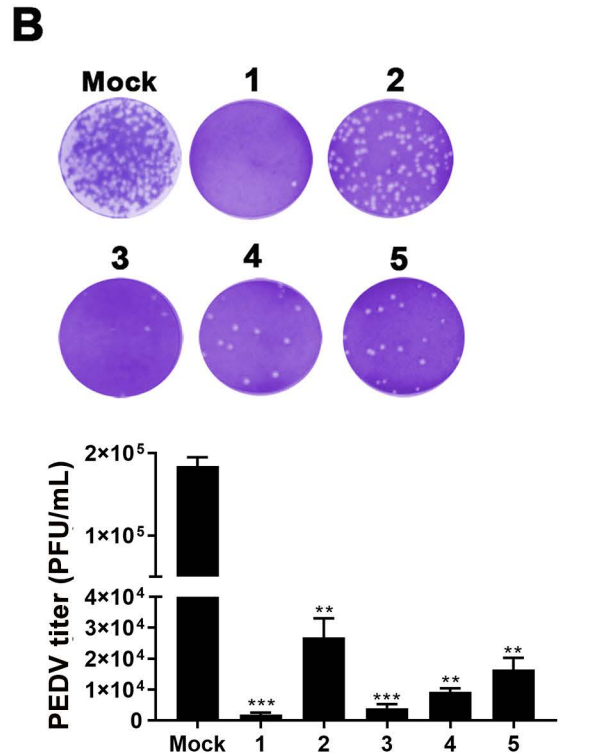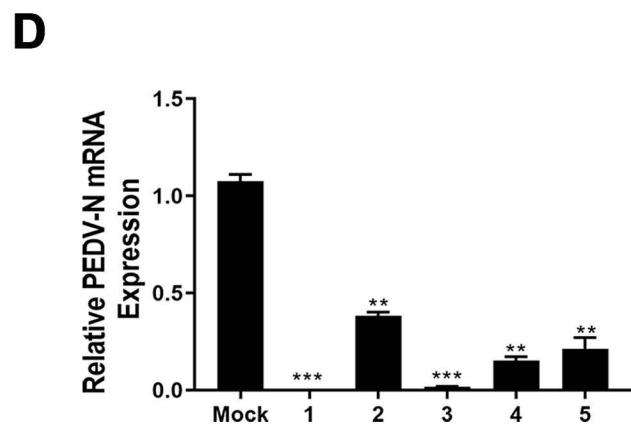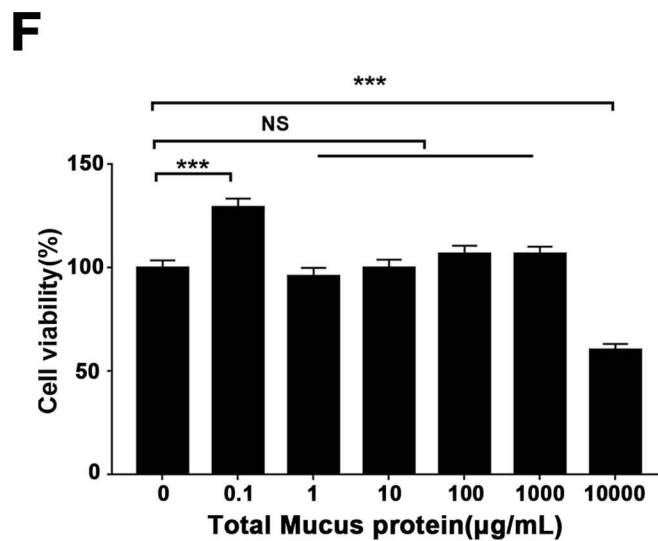

Supplement: FIG S3 [file mbio.00358-22-s0003.pdf]

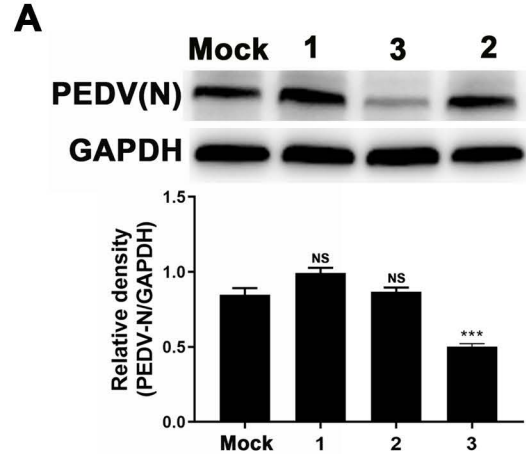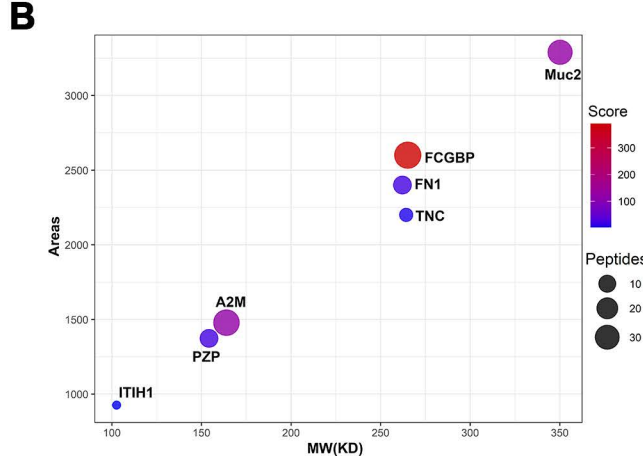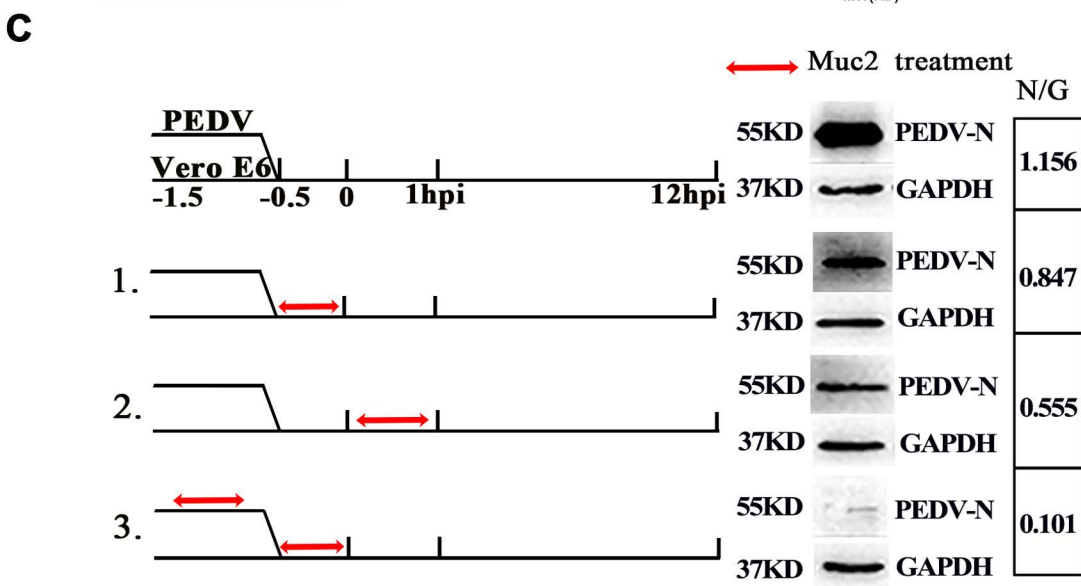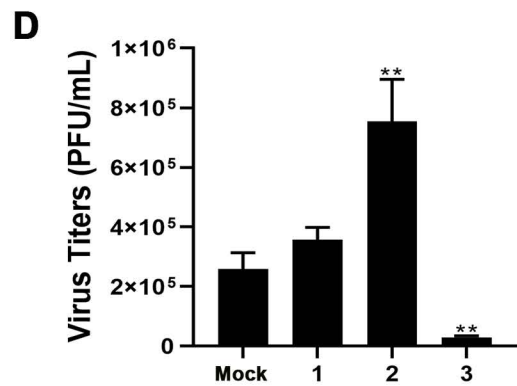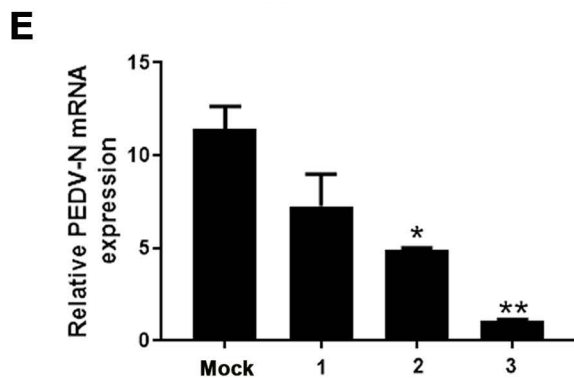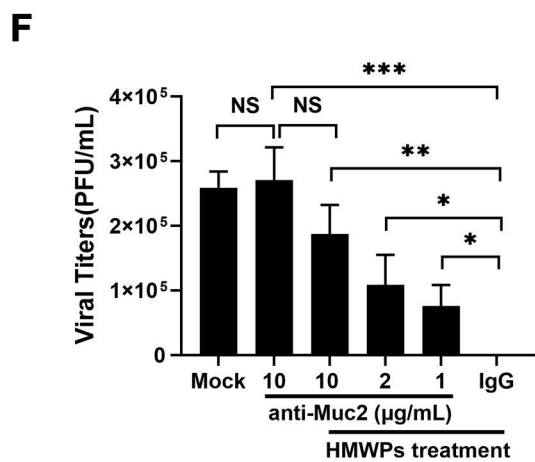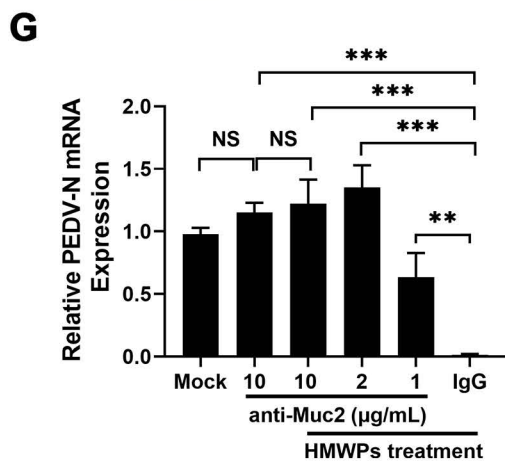

Supplement: FIG S4 [file mbio.00358-22-s0004.pdf]

**A**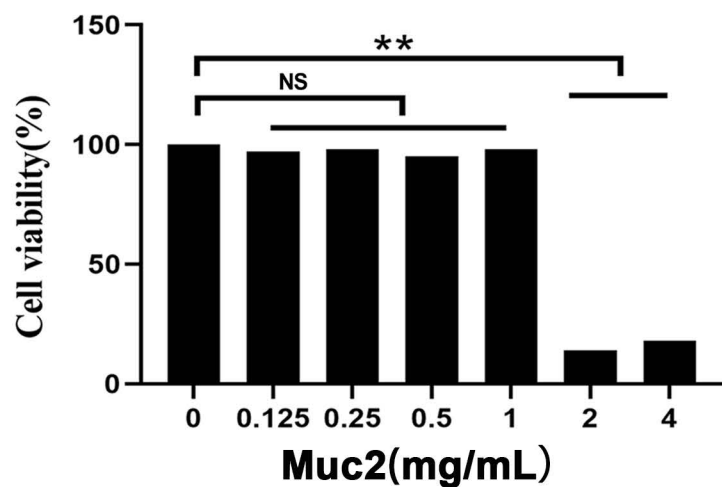**B**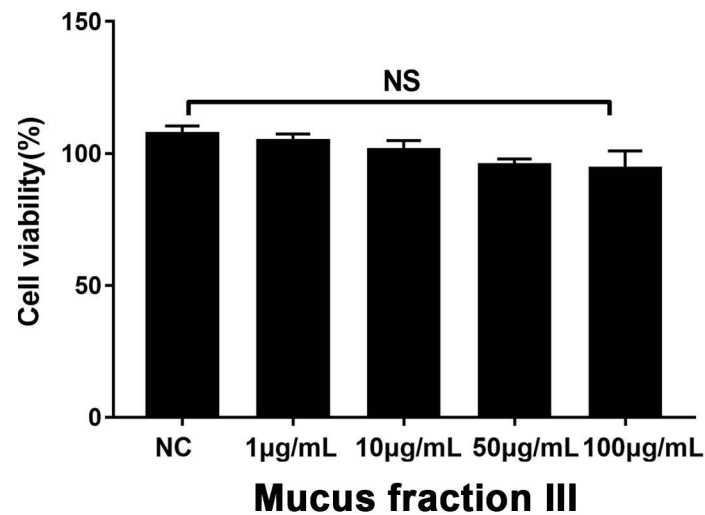**C**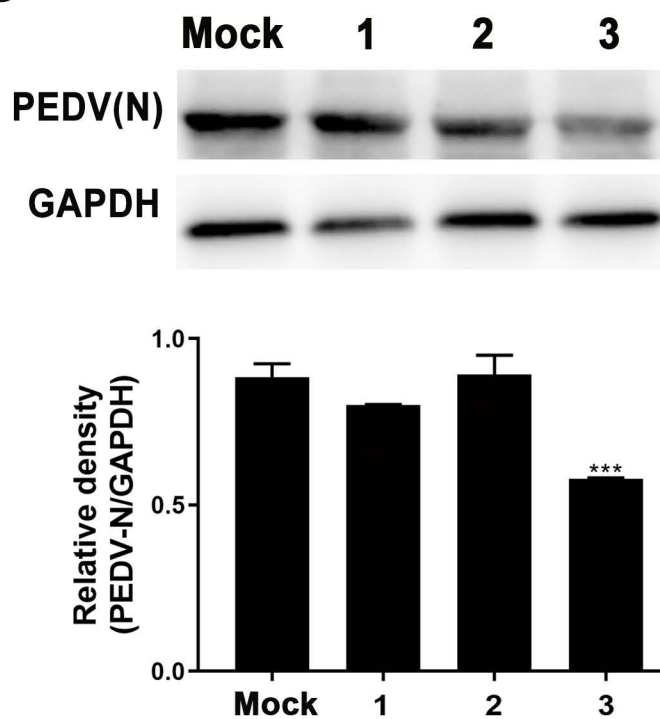**D**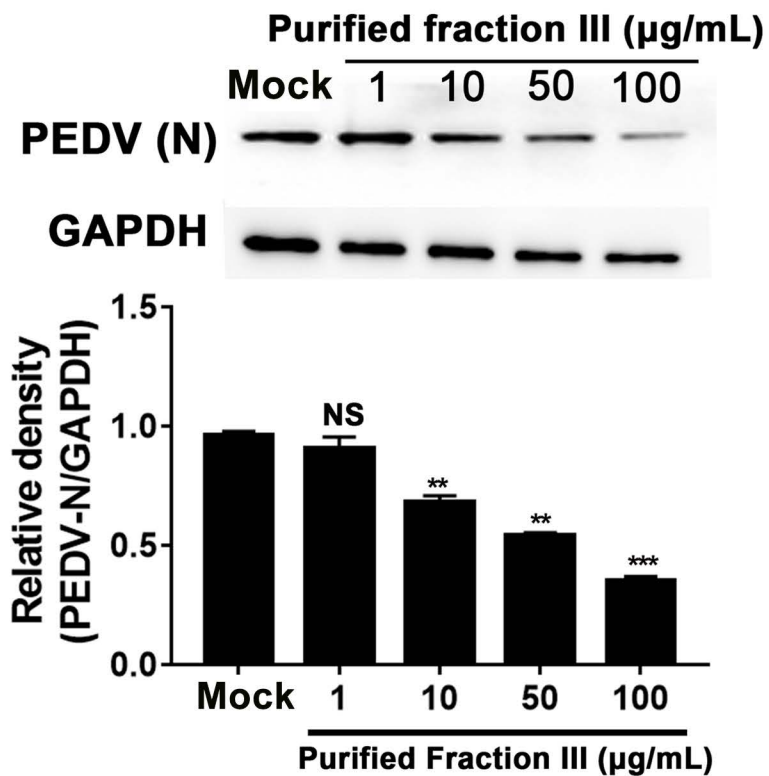

Supplement: FIG S5 [file mbio.00358-22-s0005.pdf]

**A**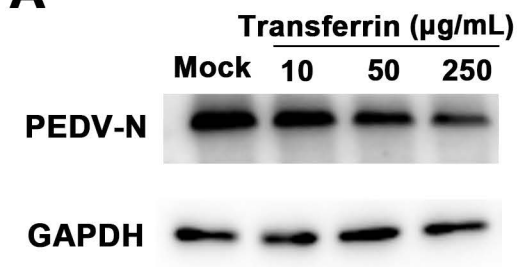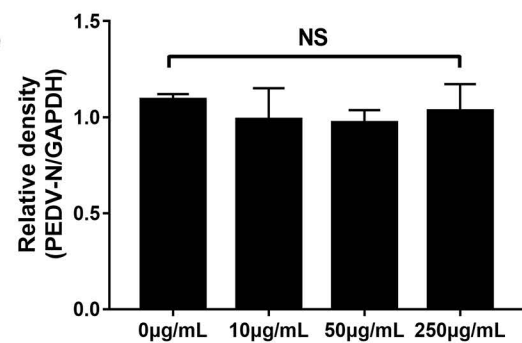**B**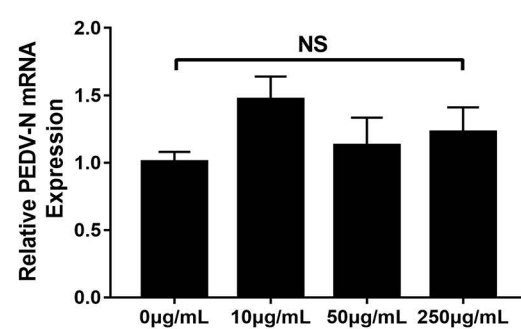**C**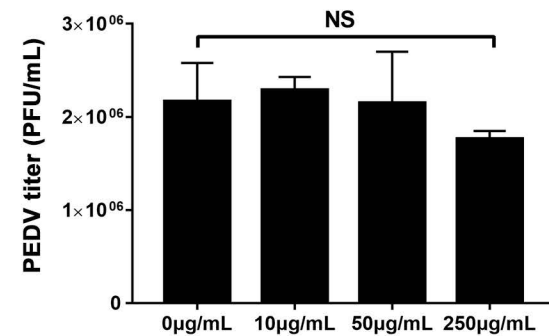**D**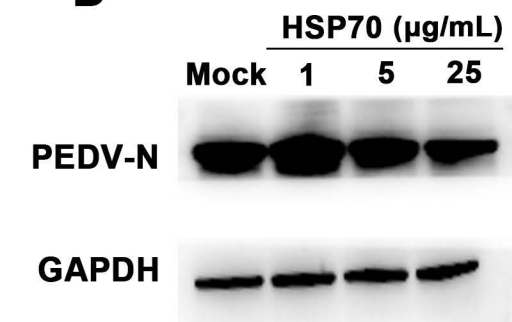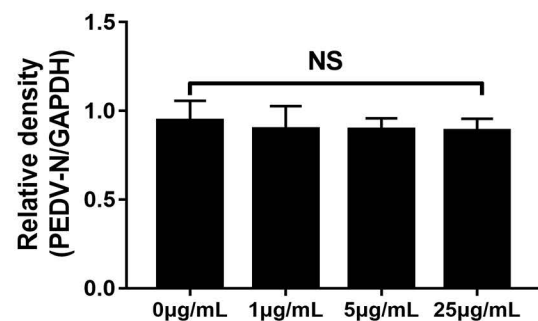**E**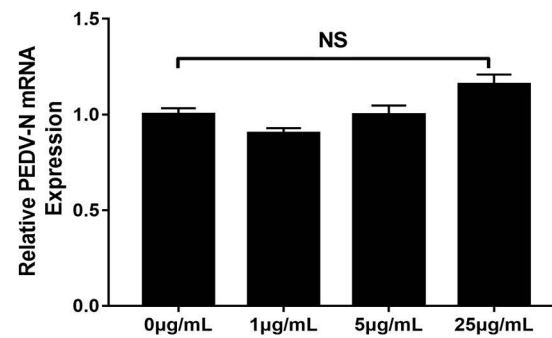**F**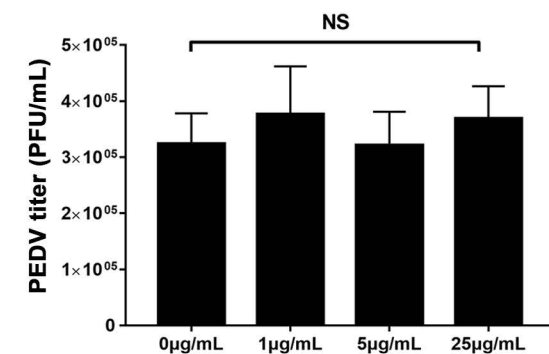**G**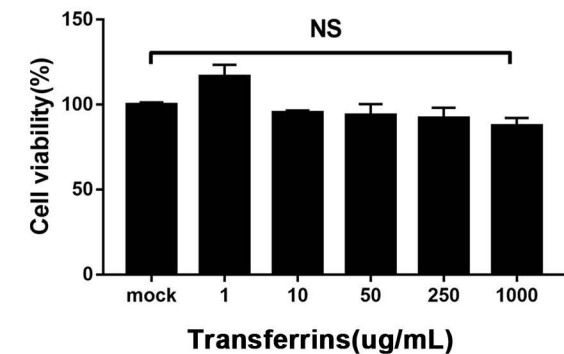**H**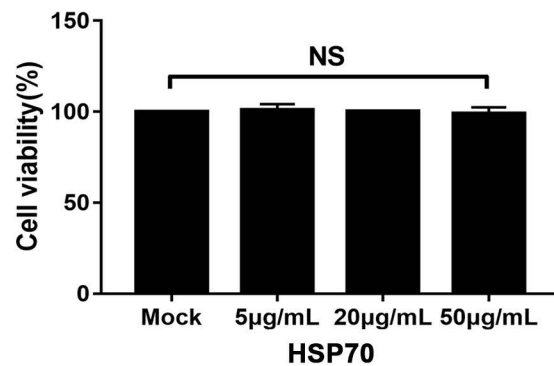**I**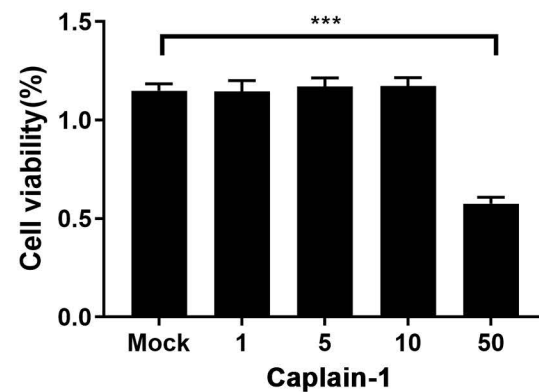**J**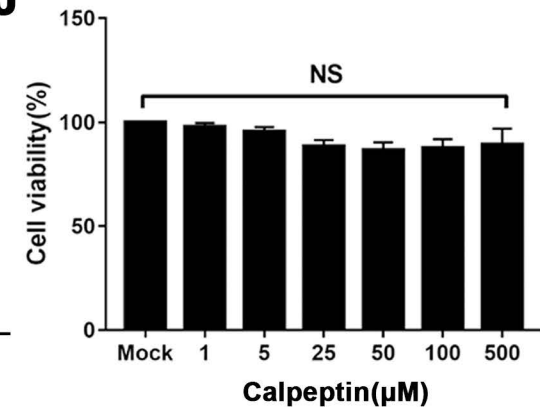

Supplement: FIG S6 [file mbio.00358-22-s0006.pdf]

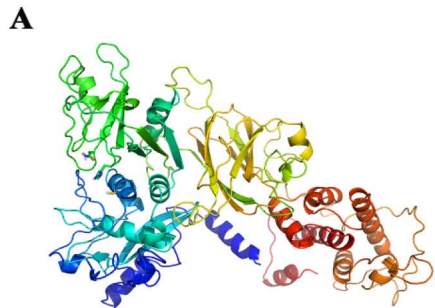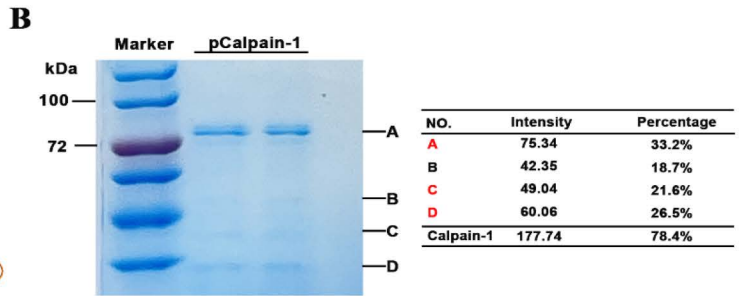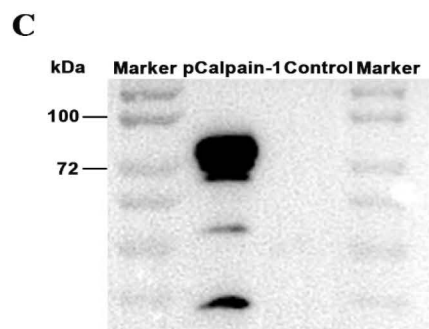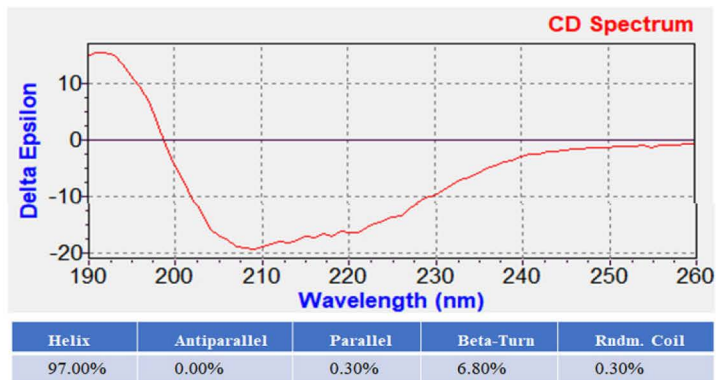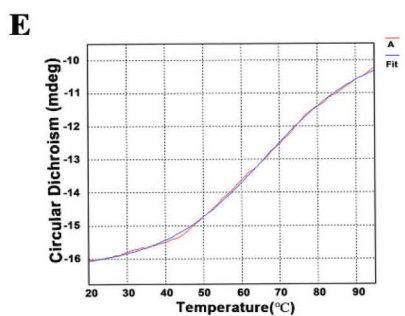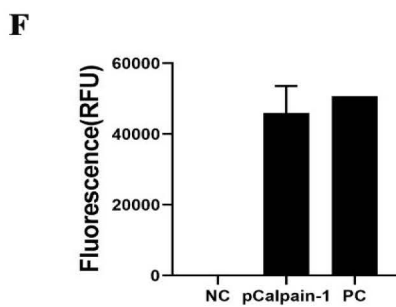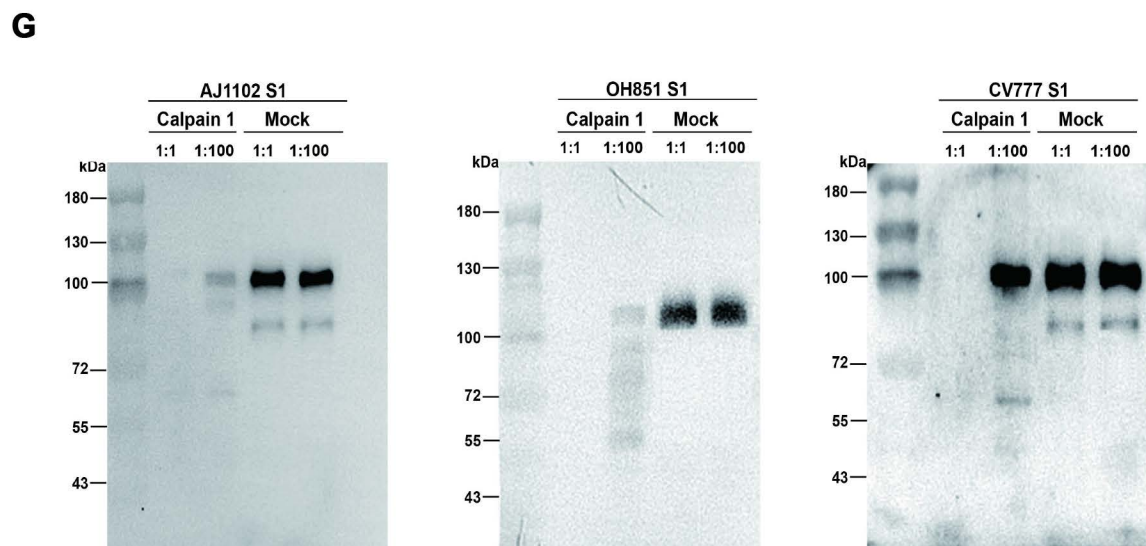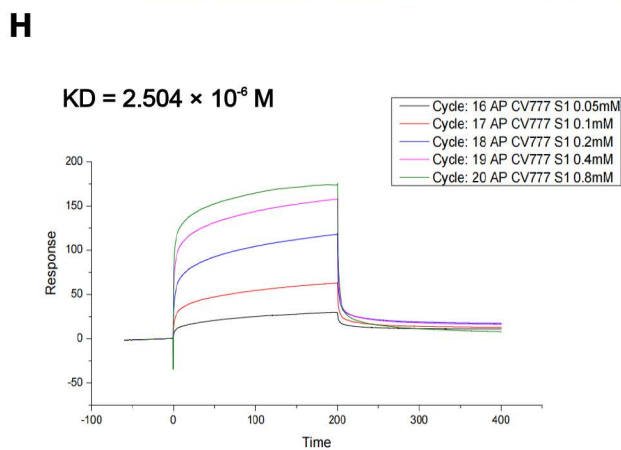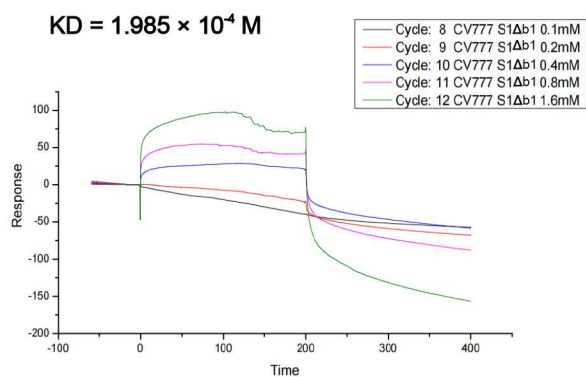

Supplement: FIG S7 [file mbio.00358-22-s0007.pdf]
