# Supplementary material for: Calpain-1: a Novel Antiviral Host Factor Identified in Porcine Small Intestinal Mucus
Source: mBio. 2022 Sep 14;13(5):e00358-22. doi: 10.1128/mbio.00358-22 (PMC9600339; doi:10.1128/mbio.00358-22)

A

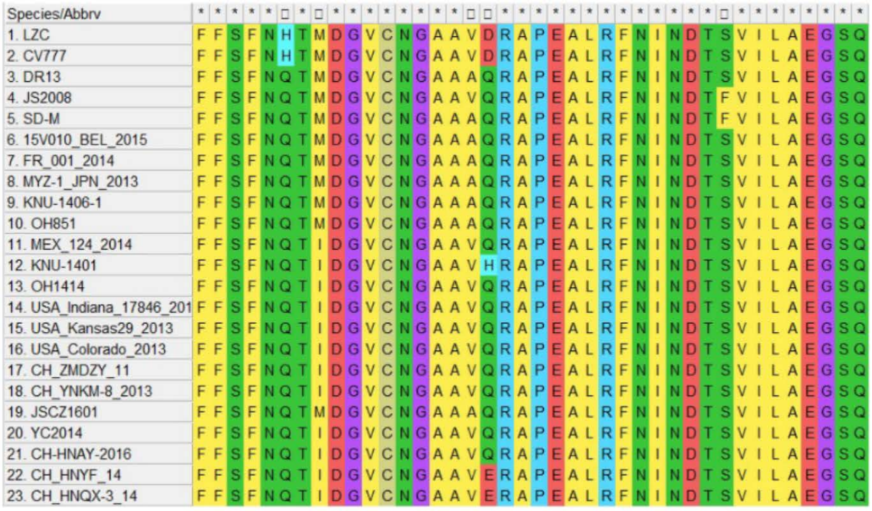

B

Percent Identity (upper triangle); Divergence (lower triangle)

	1	2	3	4	5	6	7	8	9	10	11	12	13	14	15	16	17	18	19	20	21	22	23		
1		100.0	92.5	92.5	90.0	92.5	92.5	92.5	92.5	92.5	92.5	92.5	92.5	92.5	92.5	92.5	92.5	92.5	92.5	92.5	92.5	92.5	92.5	1	LZC
2	0.0		92.5	92.5	90.0	92.5	92.5	92.5	92.5	92.5	92.5	92.5	92.5	92.5	92.5	92.5	92.5	92.5	92.5	92.5	92.5	92.5	92.5	2	CV777
3	7.9	7.9		95.0	97.5	100.0	100.0	100.0	100.0	100.0	95.0	92.5	95.0	95.0	95.0	95.0	95.0	95.0	100.0	95.0	95.0	92.5	92.5	3	DR13
4	7.9	7.9	5.2		97.5	95.0	95.0	95.0	95.0	95.0	95.0	92.5	95.0	95.0	95.0	95.0	95.0	95.0	95.0	95.0	95.0	92.5	92.5	4	JS2008
5	10.8	10.8	2.5	2.5		97.5	97.5	97.5	97.5	97.5	92.5	90.0	92.5	92.5	92.5	92.5	92.5	92.5	97.5	92.5	92.5	90.0	90.0	5	SD-M
6	7.9	7.9	0.0	5.2	2.5		100.0	100.0	100.0	100.0	95.0	92.5	95.0	95.0	95.0	95.0	95.0	95.0	100.0	95.0	95.0	92.5	92.5	6	15V010_BEL_2015
7	7.9	7.9	0.0	5.2	2.5	0.0		100.0	100.0	100.0	95.0	92.5	95.0	95.0	95.0	95.0	95.0	95.0	100.0	95.0	95.0	92.5	92.5	7	FR_001_2014
8	7.9	7.9	0.0	5.2	2.5	0.0	0.0		100.0	100.0	95.0	92.5	95.0	95.0	95.0	95.0	95.0	95.0	100.0	95.0	95.0	92.5	92.5	8	MYZ-1_JPN_2013
9	7.9	7.9	0.0	5.2	2.5	0.0	0.0	0.0		100.0	95.0	92.5	95.0	95.0	95.0	95.0	95.0	95.0	100.0	95.0	95.0	92.5	92.5	9	KNU-1406-1
10	7.9	7.9	0.0	5.2	2.5	0.0	0.0	0.0	0.0		95.0	92.5	95.0	95.0	95.0	95.0	95.0	95.0	100.0	95.0	95.0	92.5	92.5	10	OH851
11	7.9	7.9	5.2	5.2	7.9	5.2	5.2	5.2	5.2	5.2		97.5	100.0	100.0	100.0	100.0	100.0	100.0	95.0	100.0	100.0	97.5	97.5	11	MEX_124_2014
12	7.9	7.9	7.9	7.9	10.8	7.9	7.9	7.9	7.9	7.9	2.5		97.5	97.5	97.5	97.5	97.5	97.5	92.5	97.5	97.5	97.5	97.5	12	KNU-1401
13	7.9	7.9	5.2	5.2	7.9	5.2	5.2	5.2	5.2	5.2	0.0	2.5		100.0	100.0	100.0	100.0	100.0	95.0	100.0	100.0	97.5	97.5	13	OH1414
14	7.9	7.9	5.2	5.2	7.9	5.2	5.2	5.2	5.2	5.2	0.0	2.5	0.0		100.0	100.0	100.0	100.0	95.0	100.0	100.0	97.5	97.5	14	USA_Indiana_17846_2013
15	7.9	7.9	5.2	5.2	7.9	5.2	5.2	5.2	5.2	5.2	0.0	2.5	0.0	0.0		100.0	100.0	100.0	95.0	100.0	100.0	97.5	97.5	15	USA_Kansas29_2013
16	7.9	7.9	5.2	5.2	7.9	5.2	5.2	5.2	5.2	5.2	0.0	2.5	0.0	0.0	0.0		100.0	100.0	95.0	100.0	100.0	97.5	97.5	16	USA_Colorado_2013
17	7.9	7.9	5.2	5.2	7.9	5.2	5.2	5.2	5.2	5.2	0.0	2.5	0.0	0.0	0.0	0.0		100.0	95.0	100.0	100.0	97.5	97.5	17	CH_ZMDZY_11
18	7.9	7.9	5.2	5.2	7.9	5.2	5.2	5.2	5.2	5.2	0.0	2.5	0.0	0.0	0.0	0.0	0.0		95.0	100.0	100.0	97.5	97.5	18	CH_YNKM-8_2013
19	7.9	7.9	0.0	5.2	2.5	0.0	0.0	0.0	0.0	0.0	5.2	7.9	5.2	5.2	5.2	5.2	5.2	5.2		95.0	95.0	92.5	92.5	19	JSCZ1601
20	7.9	7.9	5.2	5.2	7.9	5.2	5.2	5.2	5.2	5.2	0.0	2.5	0.0	0.0	0.0	0.0	0.0	0.0	5.2		100.0	97.5	97.5	20	YC2014
21	7.9	7.9	5.2	5.2	7.9	5.2	5.2	5.2	5.2	5.2	0.0	2.5	0.0	0.0	0.0	0.0	0.0	0.0	5.2	0.0		97.5	97.5	21	CH-HNAY-2016
22	7.9	7.9	7.9	7.9	10.8	7.9	7.9	7.9	7.9	7.9	2.5	2.5	2.5	2.5	2.5	2.5	2.5	2.5	7.9	2.5	2.5		100.0	22	CH_HNYF_14
23	7.9	7.9	7.9	7.9	10.8	7.9	7.9	7.9	7.9	7.9	2.5	2.5	2.5	2.5	2.5	2.5	2.5	2.5	7.9	2.5	2.5	0.0		23	CH_HNQX-3_14
	1	2	3	4	5	6	7	8	9	10	11	12	13	14	15	16	17	18	19	20	21	22	23		

C

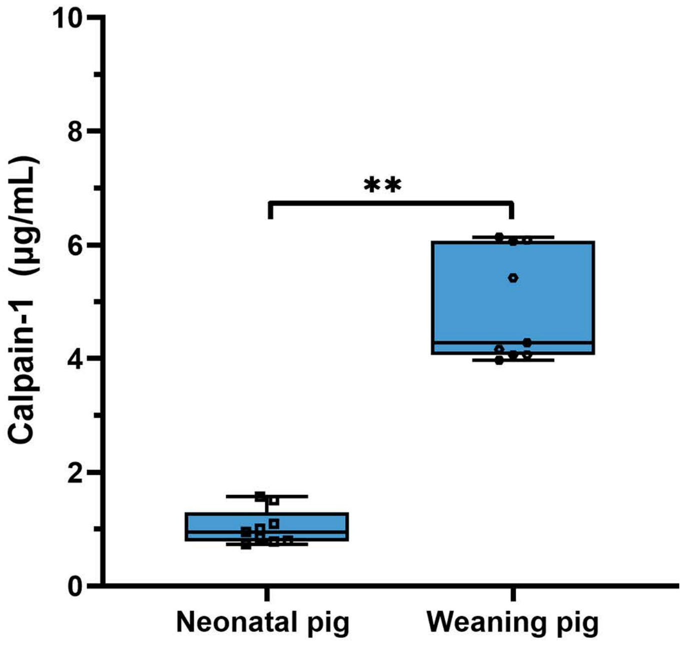

D

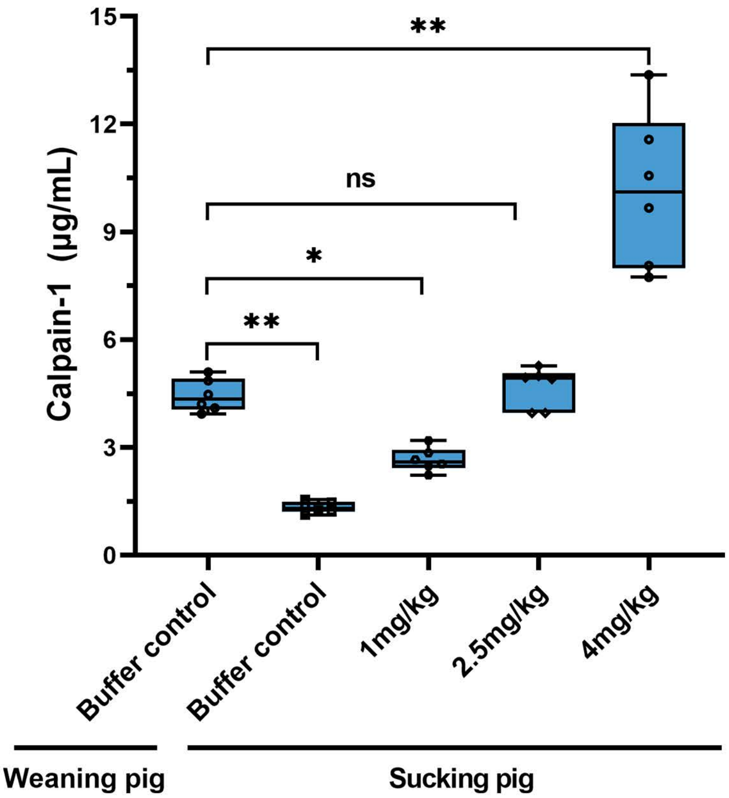

Supplement: FIG S8 [file mbio.00358-22-s0008.pdf]
