# Supplementary material for: Calpain-1: a Novel Antiviral Host Factor Identified in Porcine Small Intestinal Mucus
Source: mBio. 2022 Sep 14;13(5):e00358-22. doi: 10.1128/mbio.00358-22 (PMC9600339; doi:10.1128/mbio.00358-22)

Fig. 3L

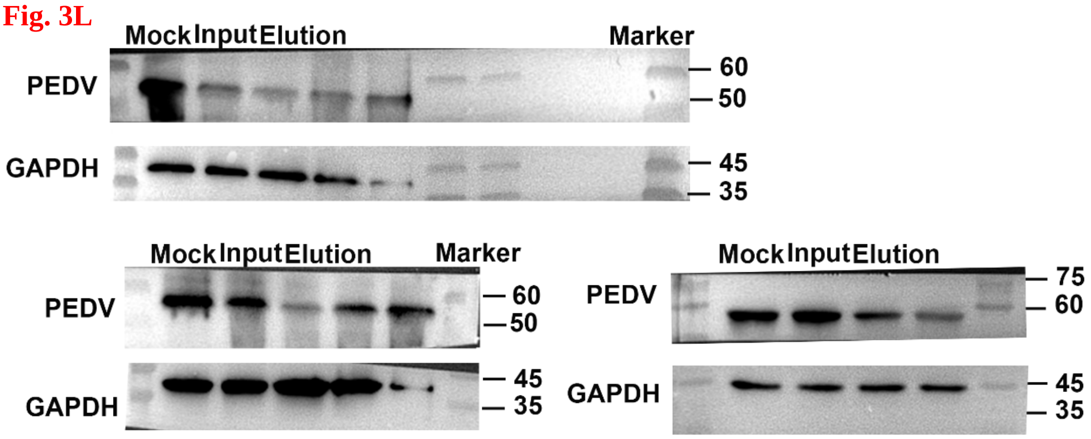

Fig. 5C

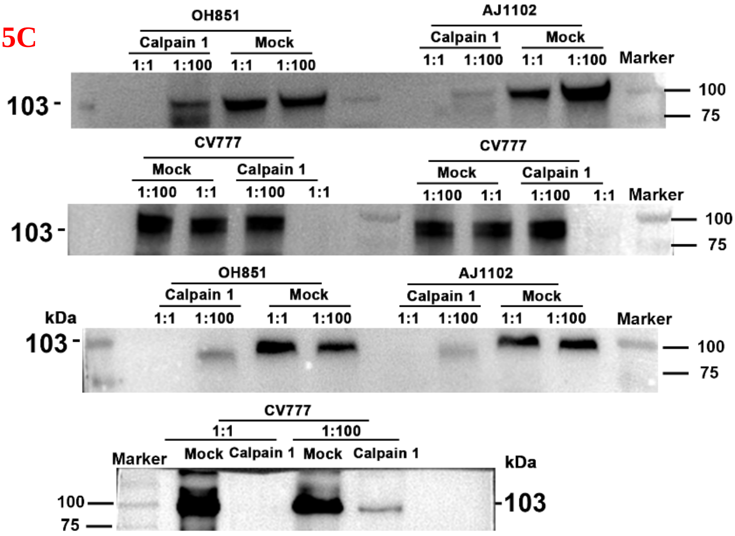

Fig. 5D

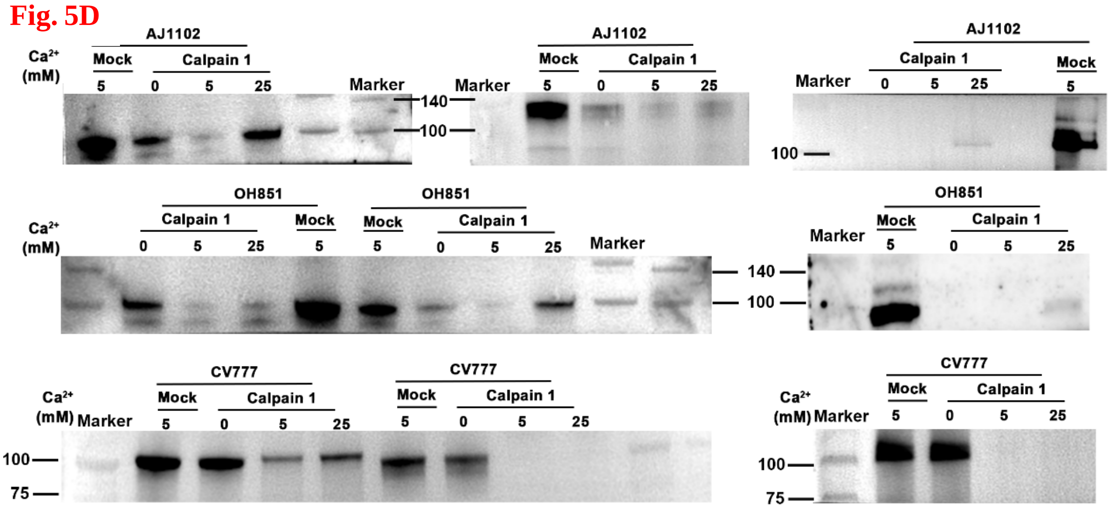

Fig. 5E

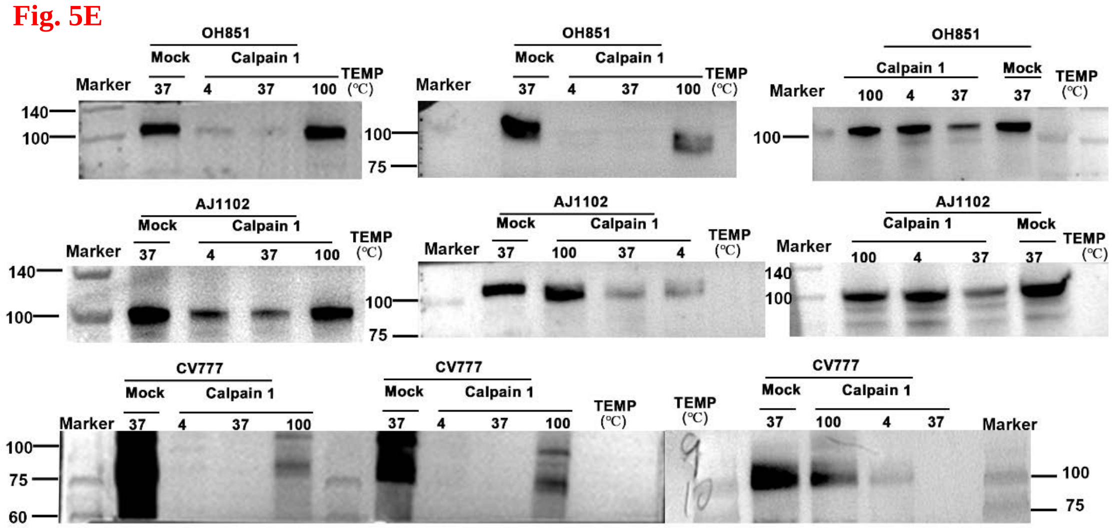

Fig. 5H

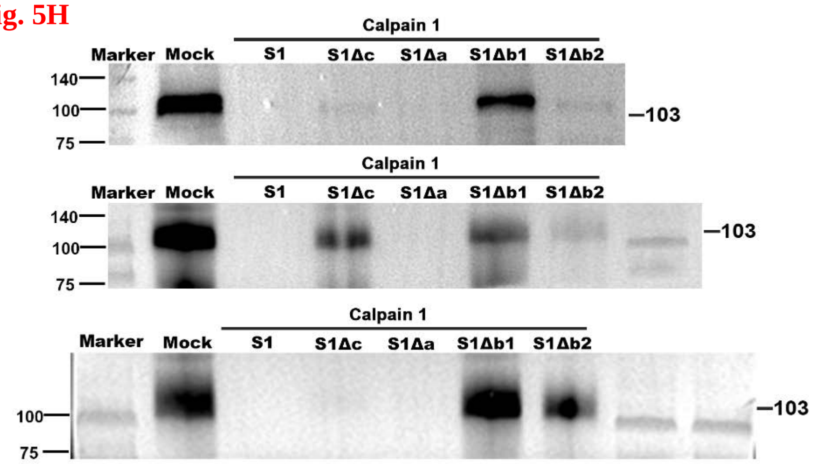

Fig. 5I

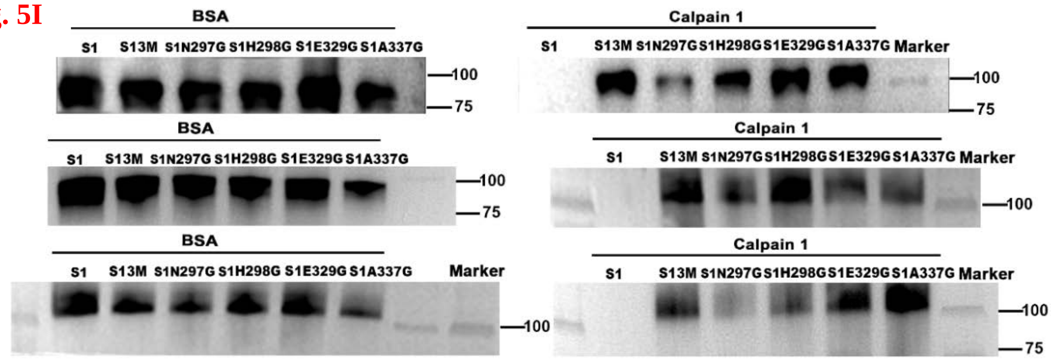

Fig. 6A

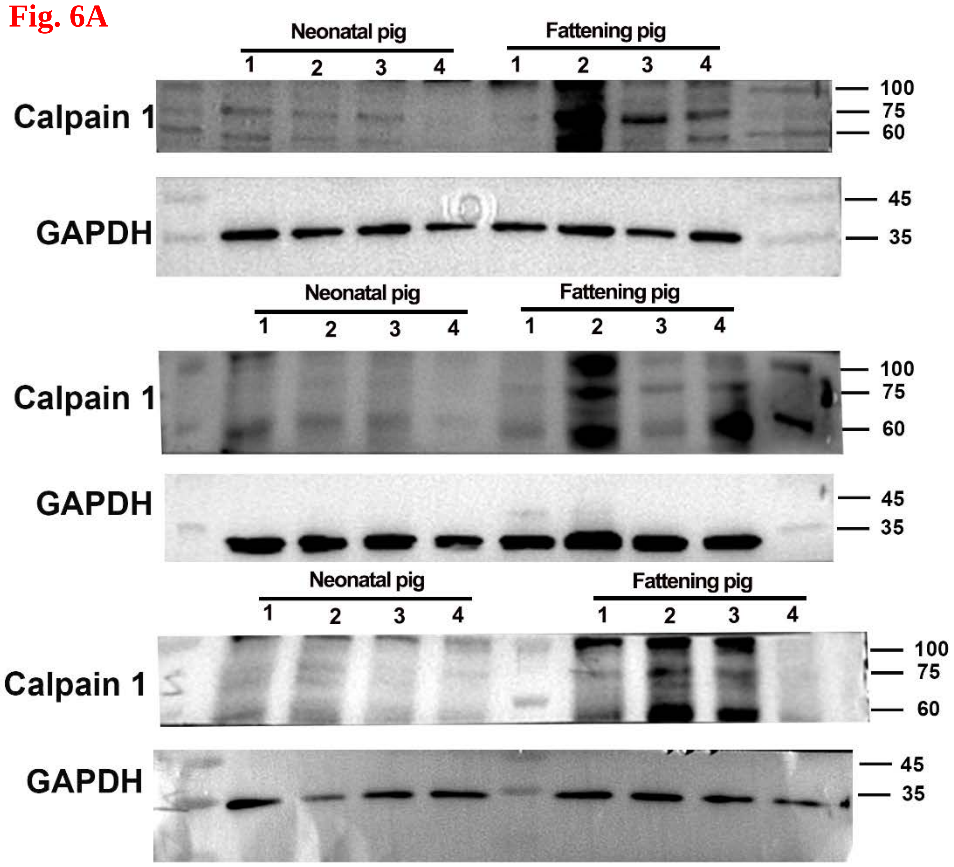
Neonatal pig
Fattening pig
1 2 3 4 1 2 3 4
Calpain 1
100
75
60
GAPDH
45
35
Neonatal pig
Fattening pig
1 2 3 4 1 2 3 4
Calpain 1
100
75
60
GAPDH
45
35
Neonatal pig
Fattening pig
1 2 3 4 1 2 3 4
Calpain 1
100
75
60
GAPDH
45
35

Fig. 7B

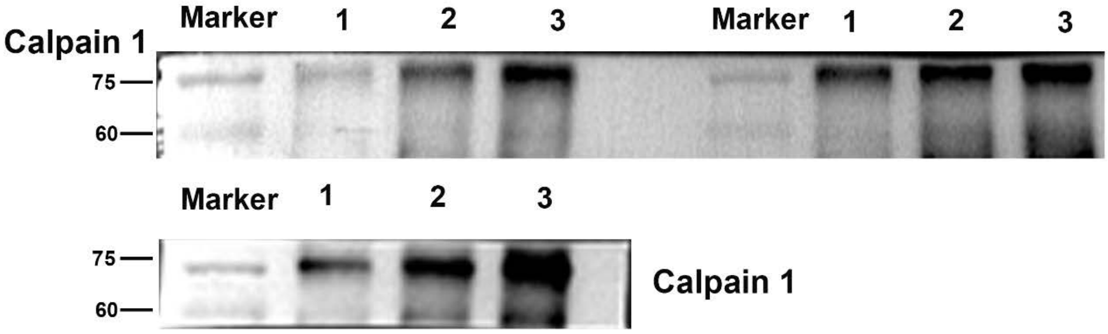
Calpain 1
Marker 1 2 3
Marker 1 2 3
75
60
Marker 1 2 3
75
60
Calpain 1

Fig. 7E

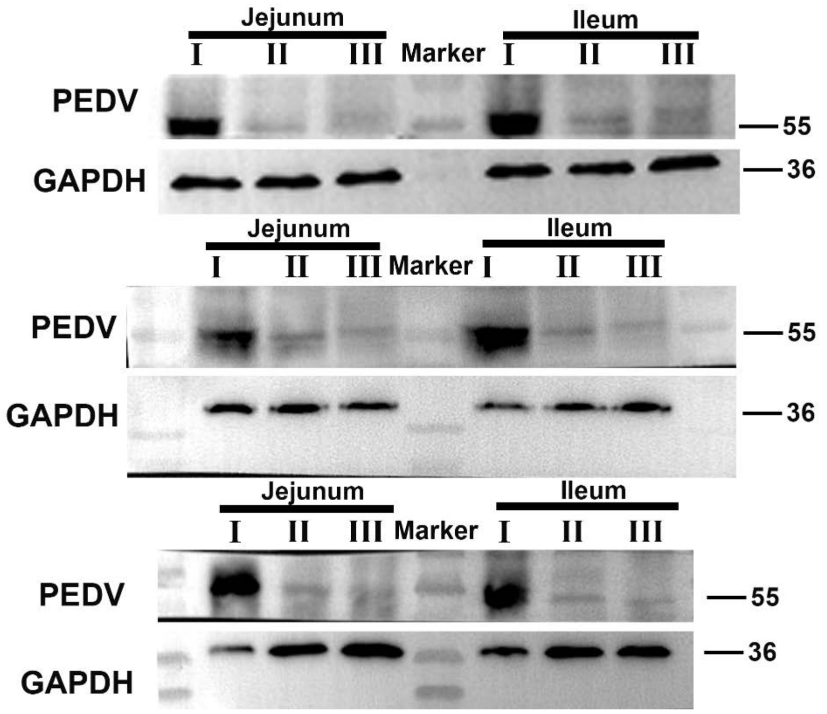

Fig.S3C

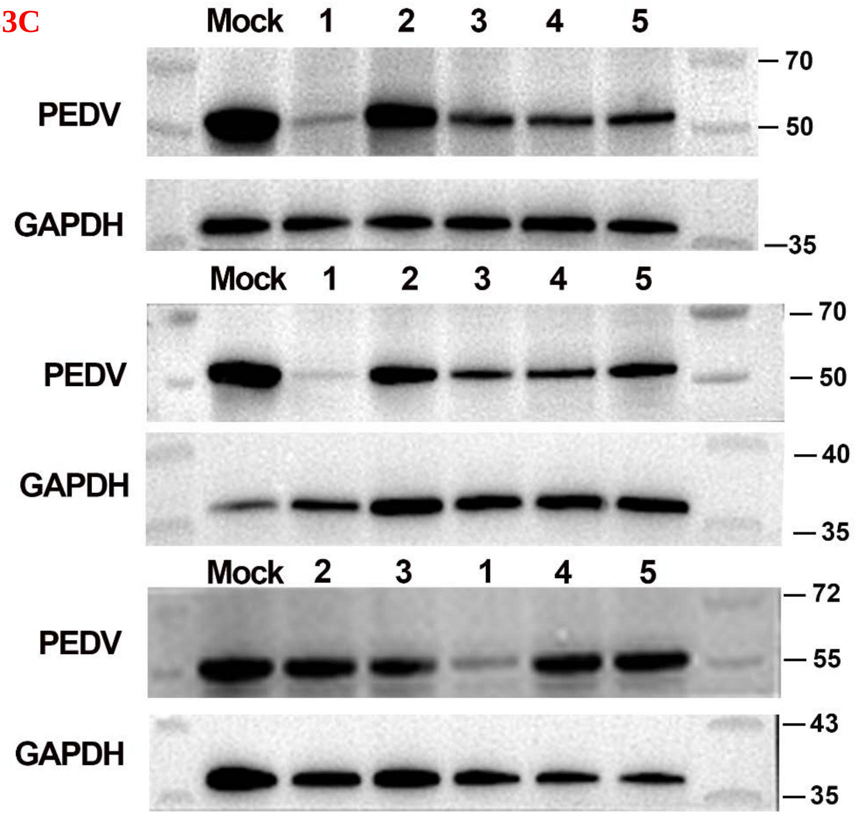

Fig. S4A

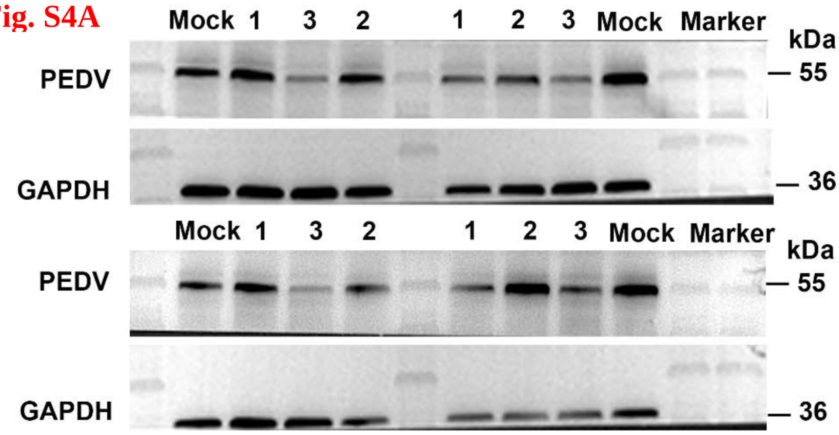

Fig. S4C

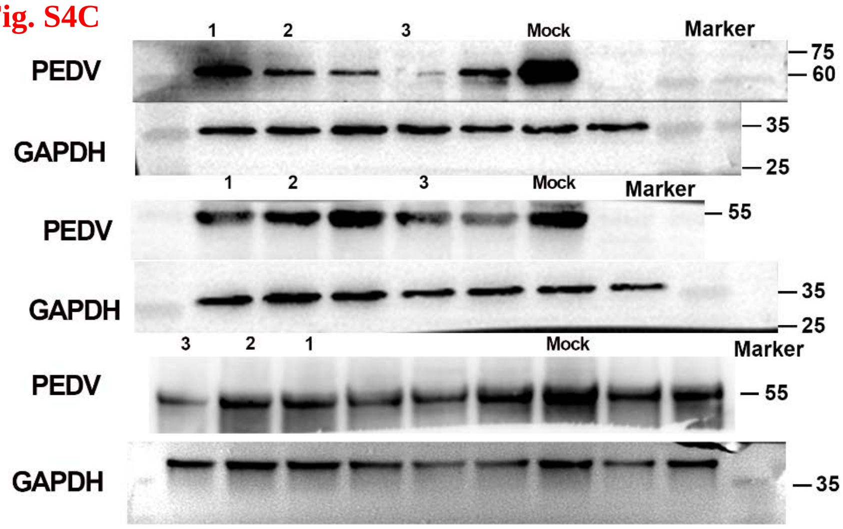

Fig. S5C

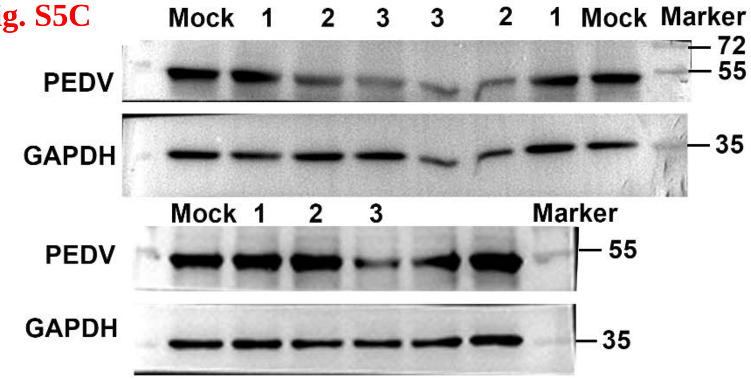

Fig. S5D

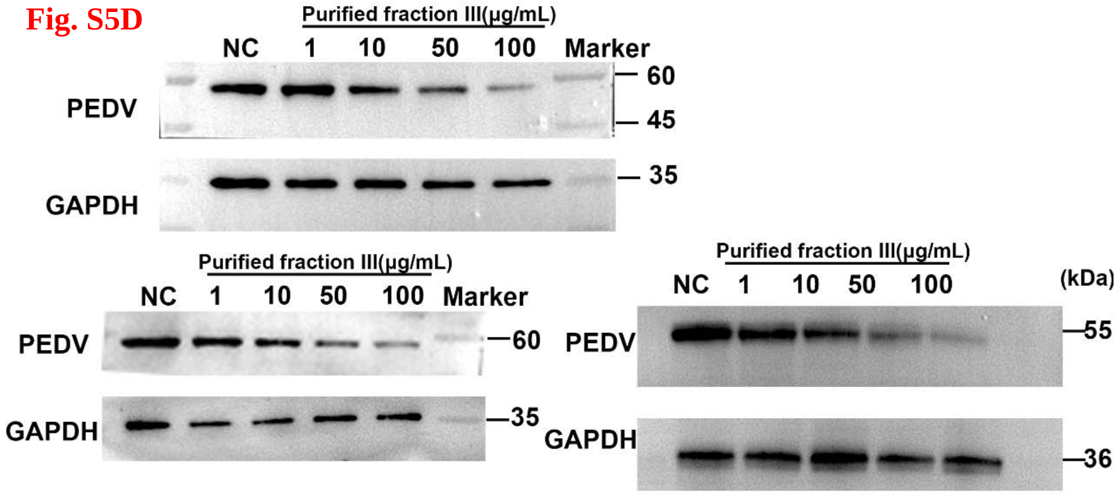

Fig.S6A

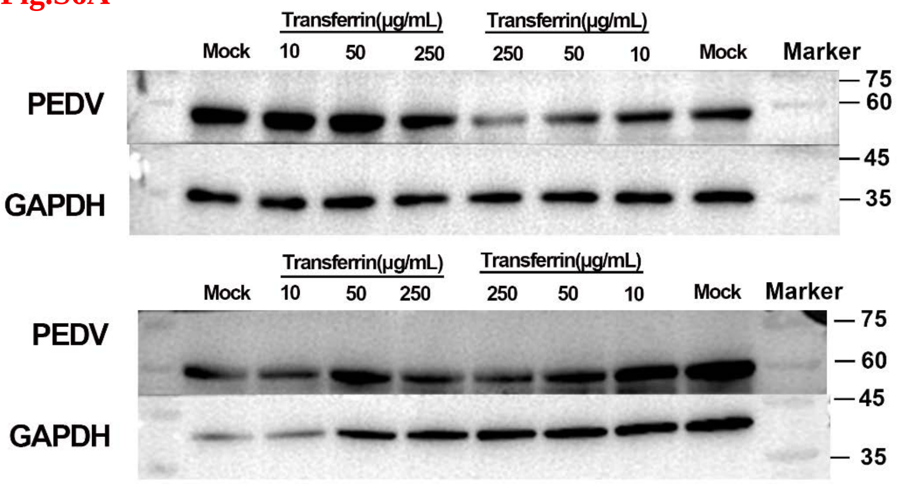

Fig.S6D

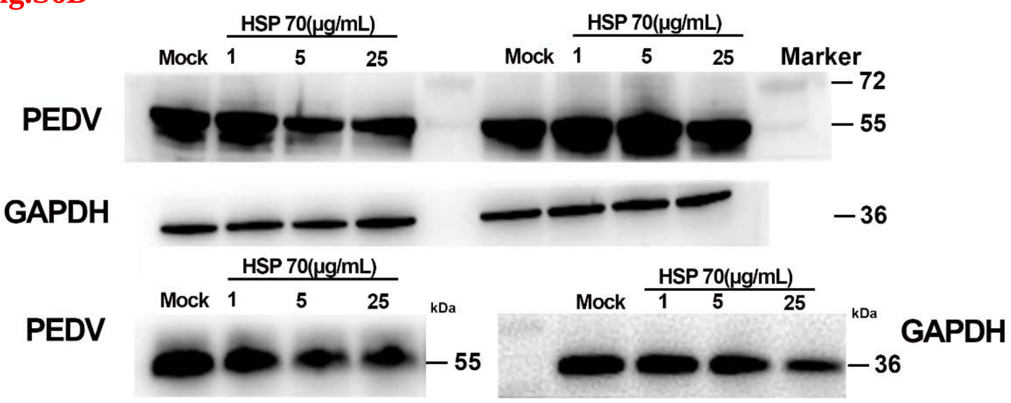

Supplement: FIG S9 [file mbio.00358-22-s0009.pdf]
